# Has1 regulates consecutive maturation and processing steps for assembly of 60S ribosomal subunits

**DOI:** 10.1093/nar/gkt545

**Published:** 2013-06-20

**Authors:** Jill A. Dembowski, Benjamin Kuo, John L. Woolford

**Affiliations:** Department of Biological Sciences, Carnegie Mellon University, Pittsburgh, PA 15213, USA

## Abstract

Ribosome biogenesis requires ∼200 assembly factors in *Saccharomyces cerevisiae*. The pre-ribosomal RNA (rRNA) processing defects associated with depletion of most of these factors have been characterized. However, how assembly factors drive the construction of ribonucleoprotein neighborhoods and how structural rearrangements are coupled to pre-rRNA processing are not understood. Here, we reveal ATP-independent and ATP-dependent roles of the Has1 DEAD-box RNA helicase in consecutive pre-rRNA processing and maturation steps for construction of 60S ribosomal subunits. Has1 associates with pre-60S ribosomes in an ATP-independent manner. Has1 binding triggers exonucleolytic trimming of 27SA_3_ pre-rRNA to generate the 5′ end of 5.8S rRNA and drives incorporation of ribosomal protein L17 with domain I of 5.8S/25S rRNA. ATP-dependent activity of Has1 promotes stable association of additional domain I ribosomal proteins that surround the polypeptide exit tunnel, which are required for downstream processing of 27SB pre-rRNA. Furthermore, in the absence of Has1, aberrant 27S pre-rRNAs are targeted for irreversible turnover. Thus, our data support a model in which Has1 helps to establish domain I architecture to prevent pre-rRNA turnover and couples domain I folding with consecutive pre-rRNA processing steps.

## INTRODUCTION

**Figure 1.**
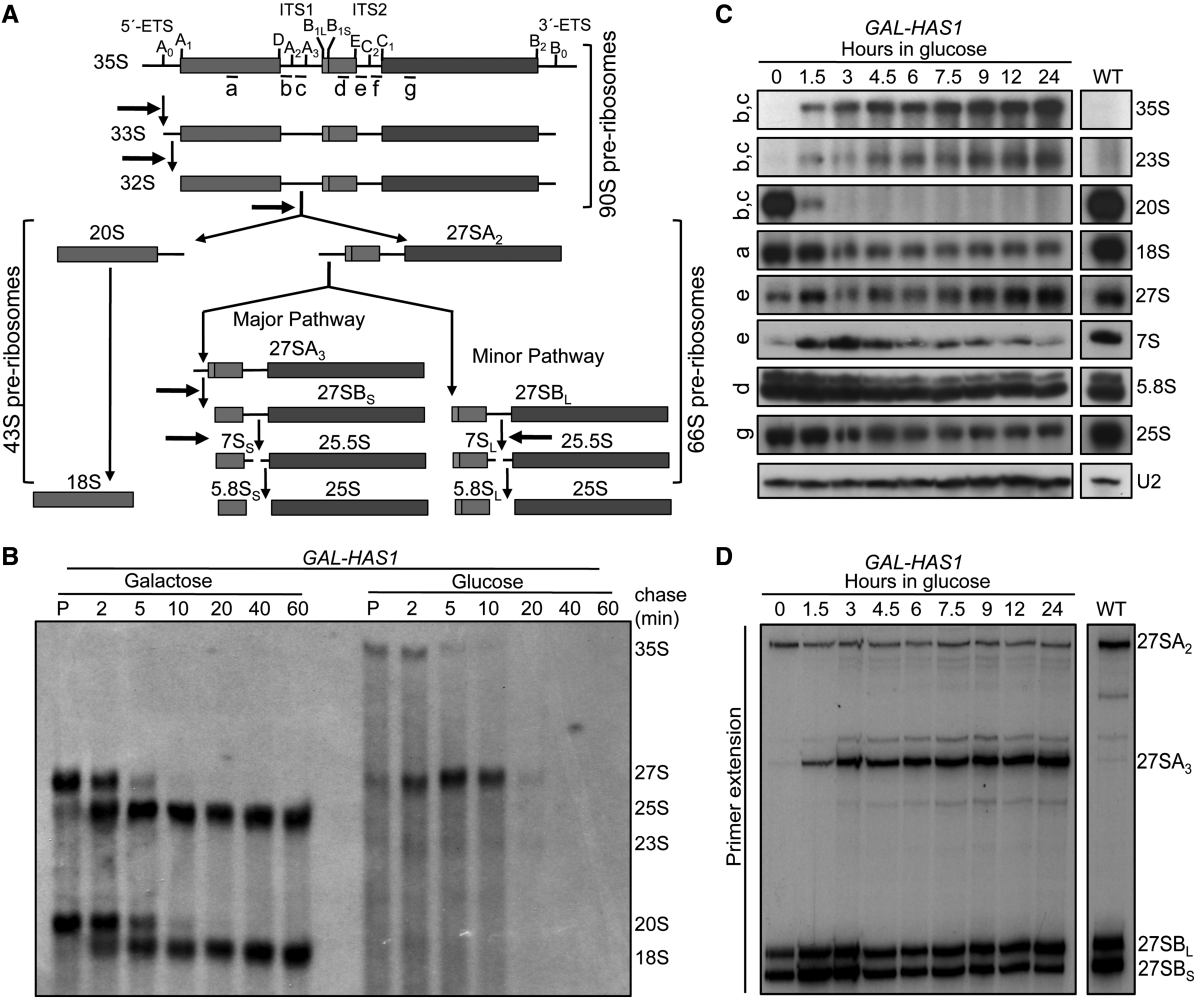
Has1 is required for processing of 35S and 27S pre-rRNAs. (**A**) Yeast pre-rRNA processing pathway. Locations of ETS and ITS sequences, processing sites (vertical ticks) and oligonucleotide probes used for northern blotting (a–g) are indicated. Processing steps blocked in the absence of Has1 are indicated by horizontal arrows. The 40S subunit contains 18S rRNA and the 60S subunit contains 5.8S, 25S and 5S rRNAs (not shown). (**B**) Pulse-chase analysis of nascent pre-rRNAs. The *GAL-HAS1* strain was grown in galactose- or glucose-containing medium lacking methionine. Cells were pulse-labeled with [methyl-^3^H]-methionine (P) and then chased with excess unlabeled methionine for 2, 5, 10, 20, 40 or 60 min followed by RNA extraction and separation on a denaturing gel. The positions of RNAs are indicated (right). (**C**) Northern blotting of cellular RNA after time-course depletion of Has1. Oligonucleotide probes (left) and RNAs detected (right) are indicated. U2 snRNA is the loading control. Wild-type (WT) cells were grown in glucose-containing medium. (**D**) Primer extension of 27S pre-rRNAs after time-course depletion. Primer extension was carried out to differentiate 27S pre-rRNA species using oligonucleotide primer f. The same RNA samples and loading were used as for northern blotting. The 27SB and 7S pre-rRNAs increase briefly after shifting from galactose- to glucose-containing medium because of the change in carbon source (1.5 and 3 h time points) (8).

Ribosome biogenesis requires folding, modification and processing of four ribosomal RNAs (rRNAs) and association of ∼80 ribosomal proteins (r-proteins) to generate mature 40S and 60S subunits of eukaryotic ribosomes (1). These events are coupled and occur in a hierarchical manner in the context of pre-ribosomes (Figure 1A, 90S, 66S and 43S pre-ribosomes). Biogenesis requires up to 200 *trans*-acting assembly factors, which include energy-consuming and processing enzymes, scaffolding and RNA-binding proteins and protein-modifying enzymes. Small nucleolar (sno) ribonucleoprotein (RNP) particles chaperone folding and mediate post-transcriptional modification of rRNA. Most of the processing steps and factors are conserved across eukaryotes, with *Saccharomyces cerevisiae* being the most studied model organism.

Pre-rRNAs undergo precise removal of internal and external transcribed spacer (ITS, ETS) sequences to generate mature rRNAs (Figure 1A). These cleavage and processing events are irreversible, suggesting that they represent points of regulatory control to ensure that only properly assembled intermediates proceed to the next step. For the most part, *trans*-acting assembly factors have been characterized by the pre-rRNA processing steps that are blocked in their absence. However, how assembly factors participate in RNP maturation, including RNA conformational changes, stable association of r-proteins with rRNA and binding and release of assembly factors with pre-ribosomes to facilitate these processing steps, has been less well characterized (2–4).

DEAD-box RNA helicases are a ubiquitous class of highly conserved enzymes that function in nearly all processes of RNA metabolism (5). DEAD-box proteins use ATP binding and hydrolysis to facilitate local RNA unwinding, strand annealing, protein displacement or stabilization of protein complexes on RNA. These dynamic proteins can act as molecular proofreaders to distinguish between correct and incorrect RNP substrates and can provide directionality to reversible reactions by coupling them to ATP turnover (6). In yeast, 19 putative RNA helicases function in ribosome biogenesis and are thought to drive proper rRNP construction or destabilize snoRNA–rRNA duplexes and incorrect pre-rRNA structures (7). Although in many instances, the processing steps that are blocked in the absence of individual DEAD-box proteins are known, their exact RNA or protein substrates and molecular functions are not well understood. Thus, the major challenge is understanding how these proteins facilitate RNP remodeling and couple proper rRNA folding and r-protein binding to specific pre-rRNA processing steps.

Has1 is an essential conserved nucleolar DEAD-box protein involved in both 40S and 60S ribosomal subunit biogenesis (8). Of the hundreds of assembly factors required to build a yeast ribosome, only Has1, Prp43 and Rrp5 have been implicated in assembly of both the 40S and 60S subunits (8–12), suggesting that these proteins can act as global regulators of ribosome production. Has1 is required for cleavages at sites A_0_, A_1_ and A_2_ for 40S subunit maturation and has been implicated in processing of 27S pre-rRNAs to generate mature 60S subunits (8). The role of Has1 in 40S subunit assembly is thought to include the release of snoRNAs from 90S pre-ribosomes (13). However, the function of Has1 in 66S pre-ribosome maturation has not been explored. We therefore sought to determine the function of Has1 in 27S pre-rRNA processing and 66S pre-rRNP maturation by investigating effects of Has1 depletion and mutations on rRNA folding, pre-rRNA processing, r-protein association and assembly factor docking with 66S pre-ribosomes.

We demonstrate that Has1 is directly involved in 60S subunit assembly and provide insight into the function of Has1 in stabilization and processing of one rRNP neighborhood. We establish a hierarchy for Has1 recruitment to 66S pre-ribosomes and demonstrate that Has1 is the last known factor to associate with pre-ribosomes to trigger processing of 27SA_3_ pre-rRNA to generate the 5′ end of 5.8S rRNA. In the absence of Has1, domain I of 5.8S/25S rRNA fails to properly form, resulting in decreased association of r-proteins with this domain and irreversible turnover of 27S pre-rRNA intermediates. On the other hand, mutations that abolish ATP-dependent activity of Has1 *in vitro* result in normal 27SA_3_ pre-rRNA processing and little turnover of 27S intermediates *in vivo*. However, r-proteins that surround the polypeptide exit tunnel are less stably associated with domain I, and downstream processing of 27SB and 7S pre-rRNAs is delayed. Therefore, our data support an ATP-independent function of Has1 to drive maturation and prevent turnover of properly assembled 27S pre-rRNA intermediates and an ATP-dependent function to stabilize the native conformation of domain I and trigger downstream pre-rRNA processing steps.

## MATERIALS AND METHODS

### Yeast strains

Yeast strains are listed in Supplementary Table S1. Strains conditional for expression from the *GAL1* promoter or expressing C-terminally TAP-tagged or 3HA-tagged proteins were generated as described previously (14,15). Yeast were grown at 30°C in YEPD (2% dextrose, 2% peptone, 1% yeast extract) or YEPgal media (2% galactose, 2% peptone, 1% yeast extract) and were harvested during mid-log phase growth. Unless indicated phenotypes were assayed after 15–16 h growth in glucose-containing medium.

To generate *has1* mutant alleles, the *S. cerevisiae HAS1* open reading frame including 500 base pairs upstream of the start codon and 300 base pairs downstream of the stop codon was cloned into pRS315. Mutations were introduced using the QuickChange II Site-Directed Mutagenesis Kit (Stratagene). Residues targeted for mutagenesis were Q69A (CAG→GCT), K92A (AAA→GCA), E197Q (GAA→CAA), S228A (TCA→GCA), T230A (ACA→GCA) and H375E (CAT→GAA) (16). Plasmids bearing mutant alleles were transformed into JWY9309 (*GAL-HA-HAS1 RPF2-TAP*) and maintained by growth on media lacking leucine. Mutant protein expression and stability were verified by western blotting using anti-Has1 antisera.

### Analysis of RNA

RNA from whole-cell lysates or purified pre-ribosomes was extracted as described previously (17,18). Steady-state levels of pre-rRNA or rRNA were analyzed by northern blotting (17) and primer extension (19). Pulse-chase analysis was carried out as described (20) with the following modifications: a 7.5 ml aliquot of yeast at OD_610_ 0.3–0.4 was pulse labeled with 198 μCi [3H-methyl]-methionine (Perkin Elmer) for 5 min, then chased with excess non-radioactive methionine at a final concentration of 5 mM. RNA was extracted from samples collected, and 20 000 cpm were separated on denaturing agarose gels followed by capillary transfer to Zeta-Probe GT blotting membranes (Bio-Rad). Blots were exposed to film using BioMax Transcreen LE Intensifying Screens (Kodak).

### Affinity purification and analysis of proteins

Whole-cell lysates were prepared from 150 to 200 ml of cultures (21) followed by affinity purification of TAP-tagged proteins and associated pre-ribosomes on 30 μl of immunoglobulin G (IgG)-conjugated Dynal beads (Life Technologies) (22). Pre-ribosomes were eluted from IgG beads by cleavage of the TAP tag with 10 U TEV Protease (Life Technologies). Eluted proteins were precipitated with 10% trichloroacetic acid (TCA) and separated on 4–20% polyacrylamide NOVEX or 4–12% NuPAGE gels (Life Technologies). Silver staining and western blotting were performed according to standard procedures. Anti-HA (Roche) and anti-TAP (Open Biosystems) antibodies were used to detect epitope-tagged proteins.

### Two-hybrid assays

Open reading frames were moved into either the pACTGW-attR or pASGW-attR two-hybrid vector (23) using the Gateway recombination-based cloning system (Life Technologies). Plasmids were transformed into PJ69-4a or PJ69-4α yeast, which were mated to test for potential interactions. Pair-wise protein–protein interactions were tested for activation of the *GAL-HIS3* or *GAL-ADE2* reporter gene as described previously (24).

### iTRAQ mass spectrometry

Cell lysates from 2 l of yeast cultures containing TAP-tagged Rpf2 were used to purify pre-ribosomes in the presence and absence of Has1 on 300 μl IgG-conjugated beads as described earlier in the text. Before TCA precipitation of proteins, each sample was separated into two tubes for duplicate iTRAQ analysis, and sample yield was verified by SDS–PAGE and silver staining. Dried pellets were sent to Penn State Hershey Core Research Facilities for trypsin digestion and labeling with iTRAQ reagents 117, 118, 119 and 121 (Applied Biosystems). Peptides were separated by 2D liquid chromatography, and parent ions were identified on a Sciex/ABI 5800 MALDI-TOF mass spectrometer. Proteins identified with >95% confidence were used for further data analysis. iTRAQ ratios as an average of all peptides for each protein were obtained using the Protein Pilot 4.0 program. For each pair-wise comparison, data were normalized to the change in ratio of the TAP-tagged protein. Normalized ratios for technical replicates were averaged and used to calculate the standard error of the mean. Processed iTRAQ data are available in Supplementary Table S2.

### Chemical probing

*In vivo* structure probing with dimethyl sulfate (DMS) was carried out as described (25), except that Transcriptor Reverse Transcriptase (Roche) was used for primer extensions with oligonucleotides designed to bind to ITS sequences within the pre-rRNA.

### PyMOL

PyMOL images of rRNA and proteins were generated using PDB files 3U5H and 3U5I (26). Pymol representation of Has1 with predicted binding sites of RNA and ATP (Figure 7B) was generated by aligning the Phyre predicted structure of Has1 (27) with Ddx19 bound to ATP and RNA (3G0H) (28). The amino acids Q69 and K92 of Has1 align with Q119 and K144 of Ddx19, respectively.

## RESULTS

### Has1 is necessary for processing of 27SA_3_ and 27SB pre-rRNAs for 60S subunit biogenesis

To begin to investigate the roles of Has1 in 60S subunit biogenesis, we assayed effects of Has1 depletion using the pAS24-*HAS1* strain (8), in which plasmid-borne *HAS1* expression was driven by the *GAL* promoter, and the chromosomal copy of *HAS1* was deleted. After shifting to glucose-containing medium to deplete Has1, we observed a deficit of 40S subunits (Supplementary Figure S1A, middle panels), consistent with previous observations. However, we did not observe complete depletion of Has1 from sucrose gradient fractions containing 66S pre-ribosomes (Supplementary Figure S1B, panels pAS24-*HAS1*), potentially masking defects in 60S subunit production. Therefore, we built a new *GAL-HAS1* strain by placing genomic *HAS1* under control of the *GAL* promoter, with the prediction that expression would be less robust for a chromosomal construct. Using this strain, we observed more complete depletion of Has1 from 66S pre-ribosomes (Supplementary Figure S1B, panels *GAL-HAS1*), resulting in both 40S and 60S subunit deficits (Supplementary Figure S1A, right panels). These data suggest that Has1 is closely associated with 66S pre-ribosomes and plays a direct role in 60S, as well as 40S subunit biogenesis.

We next examined which pre-rRNA processing steps were affected by depletion of Has1 using the genomic *GAL-HAS1* strain. Pulse-chase analysis of nascent pre-rRNAs showed that Has1 is required for both 18S and 25S rRNA production (Figure 1B). Consistent with roles in A_0_, A_1_ and A_2_ cleavages and 27S pre-rRNA processing, 20S pre-rRNA was not made and 27S pre-rRNA was not processed in the absence of Has1. Previously, partial depletion of Has1 from 66S pre-ribosomes resulted in delayed processing of 27S pre-rRNA to 25S rRNA (8). Here, more complete depletion of Has1 resulted in delayed 27S pre-rRNA processing followed by turnover, supporting a dose-dependent effect of Has1 depletion on the stability of 27S pre-rRNA intermediates.

Northern blotting and primer extension of total cellular RNA were used to determine the effects of Has1 depletion on steady-state levels of pre-rRNA and rRNA. Between 1.5 and 3 h after shifting from galactose- to glucose-containing medium, Has1 protein dropped below endogenous levels (Supplementary Figure S1C). At this time point, we observed a dramatic decrease in 20S pre-rRNA, which correlated with increased amounts of 35S and 23S pre-rRNAs and decreased 18S rRNA and 27SA_2_ pre-rRNA (Figure 1C and D). The 23S pre-rRNA extends from the unprocessed 5′ end of 35S pre-rRNA to the A_3_ site. Thus, Has1 is necessary for cleavage at sites A_0_, A_1_ and A_2_, but not A_3_. After Has1 depletion, 27SA_3_ and 27SB_L_ pre-rRNAs accumulated and 27SB_S_ and 7S pre-rRNAs, as well as 25S and 5.8S rRNAs decreased. Thus, the 27SA_3_ pre-rRNA that was made by direct cleavage at the A_3_ site was not processed to 27SB_S_ pre-rRNA, and the 27SB_L_ pre-rRNA that was generated by direct cleavage at the B_1L_ site was not processed to 5.8S and 25S rRNAs. Therefore, Has1 is required for several pre-rRNA processing steps for both 40S and 60S subunit biogenesis (Figure 1A, horizontal arrows).

### Has1 associates with 90S, 43S and 66S pre-ribosomes

**Figure 2.**
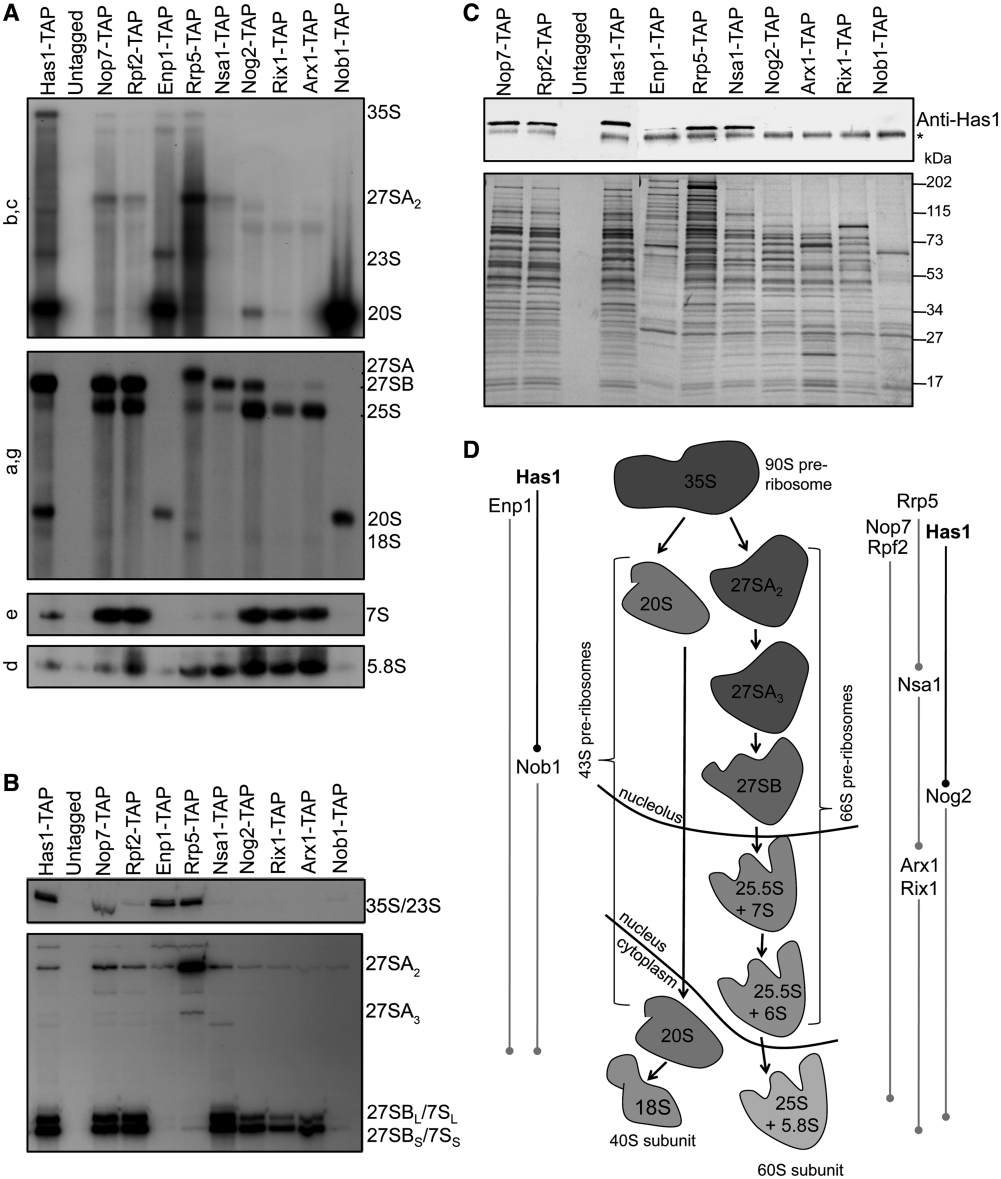
Has1 copurifies with 90S, 66S and early 43S pre-ribosomes. (**A**) Northern blotting of purified RNAs. One-step purification of pre-ribosomal particles was carried out using TAP-tagged assembly factors or an untagged control strain (top). Associated RNAs (right) were identified by northern blotting with probes a-g (left) as in Figure 1C. (**B**) Primer extension of purified RNAs. Primer extension was carried out on purified RNA samples from A using primer e. Associated RNAs are indicated (right). (**C**) Successive pre-ribosomal particles were analyzed for association with Has1. Western blotting was carried out with the anti-Has1 antibody (top). The asterisk indicates IgG stripped from beads during purification. A silver stained SDS–PAGE gel is shown to compare the relative yields of individual purifications (bottom). Molecular weight markers are shown (right). (**D**) Experimentally verified timing of assembly factor association for 43S (left) and 66S (right) pre-ribosomes.

To carefully characterize the timing of Has1 entry and exit from pre-ribosomes, we used TAP-tagged Has1 to affinity purify pre-rRNPs and identified associated RNAs (Figure 2A and B). TAP-tagged assembly factors that copurify successive assembly intermediates were used as controls (Figure 2A, B and D). Has1-TAP copurified 35S, 27SA_2_, 27SA_3_ and 27SB pre-rRNAs above background levels, consistent with Has1 being present in 90S and 66S pre-ribosomes (29,30). Because 27SA_3_ pre-rRNA is not abundant, comparatively less of this intermediate copurified with Has1-, Nop7-, Rpf2- and Nsa1-TAP (Figure 2B). We also found that Has1 copurified with 20S pre-rRNA. In support of this, we detected low levels of Has1 in 43S-containing fractions from sucrose gradients (Supplementary Figure S1B, panels wild-type).

As an orthogonal approach to pinpoint Has1 association with regard to other assembly factors, we assayed for Has1 copurification with assembly factors found in successive pre-ribosomes (Figure 2C and D). Has1 associated with 90S (Rrp5-TAP), early to middle 66S (Nop7-, Rpf2- and Nsa1-TAP) and 90S/43S particles (Enp1-TAP). The relative amount of Has1 associated with Enp1-TAP was significantly lower than that found in other particles, suggesting that Has1 and Enp1 are only present in the same particle for a short period or that Has1 is more weakly associated with Enp1-TAP particles. Has1 did not copurify with late 66S (Nog2-, Arx1- or Rix1-TAP) or late 43S (Nob1-TAP) particles. These data confirm that Has1 joins 90S pre-ribosomes and briefly travels with early 43S pre-ribosomes before Nob1 entry. Furthermore, Has1 associates with 66S pre-ribosomes and exits on or before Nog2 entry. We verified that Has1 and Nog2 are mutually exclusive for association with 66S pre-ribosomes (Supplementary Figure S2), even though they both copurify 27SB pre-rRNA (Figure 2A and B). Thus, Has1 associates with all of the early pre-rRNA intermediates in the nucleolus and may have multiple binding sites to facilitate separate functions in 40S and 60S ribosomal subunit biogenesis.

### Has1 is the last known factor to associate with 66S pre-ribosomes for processing of 27SA_3_ pre-rRNA and recruits proteins for downstream processing of 27SB pre-rRNAs

To better understand the role of Has1 in successive 27S pre-rRNA processing steps, we determined the hierarchy of Has1 recruitment to 66S pre-ribosomes relative to other factors involved in processing of 27SA_3_ and 27SB pre-rRNAs. Previously, we characterized a group of six assembly factors (Nop7, Ytm1, Erb1, Rlp7, Cic1, Nop15) that are interdependent for association with 66S pre-ribosomes and required for processing of 27SA_3_ pre-rRNA. In the absence of these ‘A_3_ factors’, there is a decrease in association of Has1 with 66S pre-ribosomes (18). R-protein L8 is required for association of A_3_ factors with 66S pre-ribosomes and processing of 27SA_3_ pre-rRNA, but does not depend on the A_3_ factors to enter pre-rRNPs (31). The presence of A_3_ factors is necessary for assembly of r-proteins L17, L26, L35 and L37 with pre-ribosomes (18), which are required for efficient cleavage at the C_2_ site in 27SB pre-rRNA (25,32). All of these factors must be in place for association of Nsa2 and Nog2 with pre-ribosomes, which are required for cleavage of 27SB pre-rRNA (18,25,31,32). Nog2 is the last factor known to enter pre-ribosomes before 27SB pre-rRNA processing (33).

**Figure 3.**
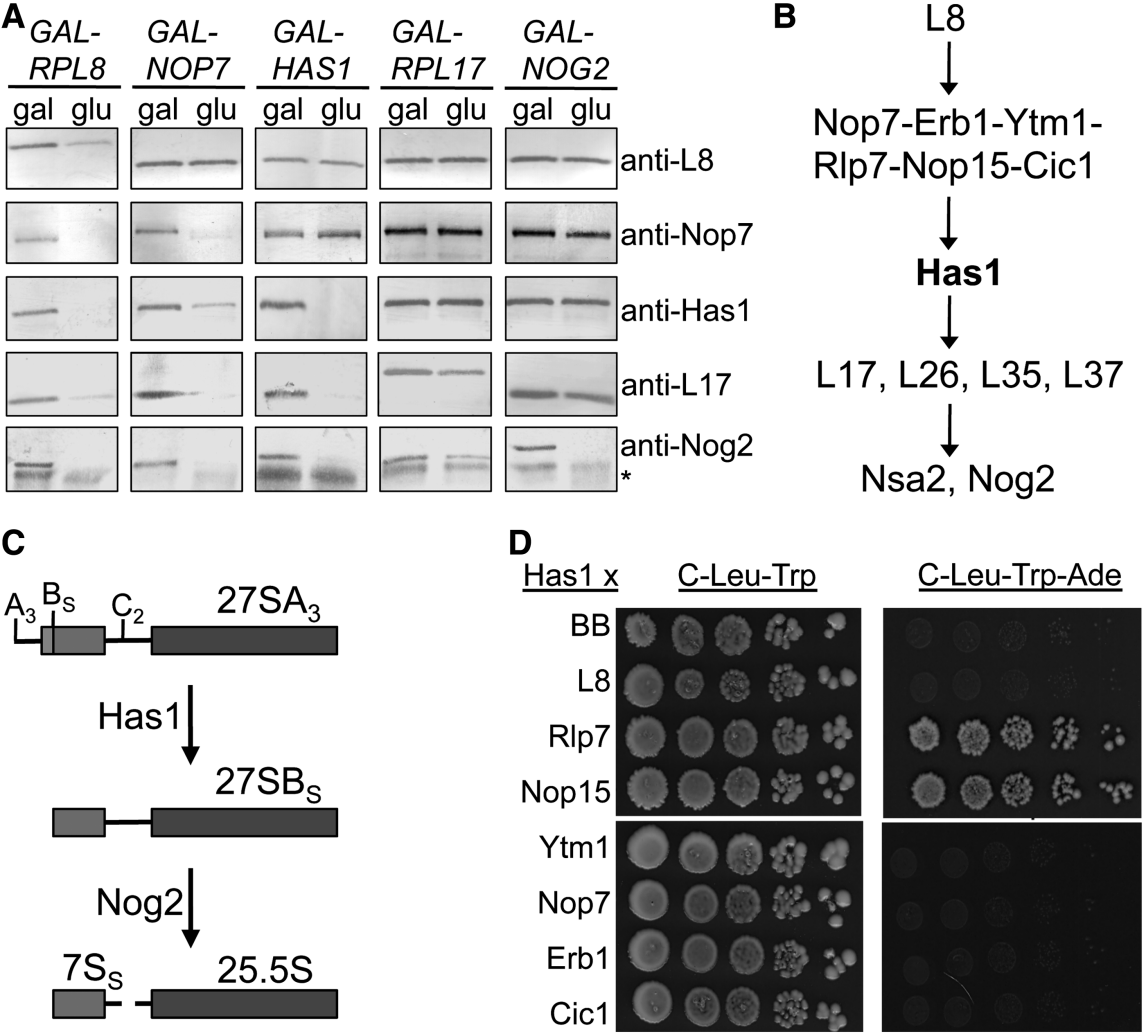
Has1 is recruited to pre-ribosomes by factors required for 27SA_3_ pre-rRNA processing. (**A**) Systematic depletion of r-proteins and assembly factors (top) followed by western blotting of proteins in 66S pre-ribosomes (right) reveals the hierarchy of protein recruitment. Rpf2-TAP (for *GAL-RPL8, GAL-NOP7 and GAL-HAS1*) or Nop7-TAP (for *GAL-RPL17 and GAL-NOG2*) were used to purify 66S pre-ribosomes and assay for changes in protein association. The asterisk indicates IgG stripped from beads during purification. (**B**) The hierarchy of protein association to 66S pre-ribosomes with respect to Has1. Commas indicate that proteins are not interdependent, and hyphens indicate that proteins are interdependent for recruitment to pre-ribosomes. (**C**) Has1 and Nog2 are the last known factors to be recruited to pre-ribosomes to trigger consecutive 27S pre-rRNA processing steps. (**D**) Two-hybrid assays reveal potential protein–protein interactions between Has1 and Rlp7 or Nop15. The 10-fold serial dilutions of yeast were spotted onto control (C-Leu-Trp) and experimental (C-Leu-Trp-Ade) medium. Positive protein interactions activate transcription of a reporter gene, which allows yeast to grow in the absence of adenine (Ade). BB, plasmid backbone.

We depleted representative factors from each of these groups and asked how protein recruitment to 66S pre-ribosomes was affected (Figure 3A and B). When L8 was depleted, Nop7, Has1, L17 and Nog2 did not join 66S pre-ribosomes, placing L8 at the beginning of the recruitment pathway. When Nop7 was depleted, L8 did associate, but Has1, L17 and Nog2 did not, placing Nop7 and the other interdependent A_3_ factors next in the pathway. Furthermore, when Has1 was depleted, L8 and Nop7 entered, but L17 and Nog2 did not. When L17 was depleted, L8, Nop7 and Has1 entered but Nog2 did not, and when Nog2 was depleted, all other factors entered pre-ribosomes. Because Has1 is the last known factor in this assembly hierarchy that is required for 27SA_3_ pre-rRNA processing, we hypothesize that it plays a key role in organizing pre-rRNPs or recruiting exonucleases to trigger this step (Figure 3C). Furthermore, the defect in C_2_ cleavage observed in the absence of Has1 may reflect a role in recruiting L17, L26, L35 and L37 or Nog2 to pre-ribosomes.

We next asked if Has1 binds to any of the upstream factors on which it depends to enter pre-ribosomes. These include potential recruiters of Has1. We tested for two-hybrid interactions of Has1 with L8, Rlp7, Nop15, Ytm1, Nop7, Erb1 and Cic1 (Figure 3D). Has1 displayed positive interactions with Nop15 and Rlp7. Taken together, these data provide a model for the hierarchy of assembly factor and r-protein recruitment to 66S pre-ribosomes with respect to Has1 and support a means by which Has1 may be recruited to pre-ribosomes by Rlp7 and/or Nop15.

### Early pre-ribosomal intermediates are enriched after Has1 depletion

To further investigate the role of Has1 in organizing pre-ribosomes, we determined how the protein composition of consecutive pre-ribosomal intermediates changed in the absence of Has1. We purified total 66S pre-ribosomes with Nop7- and Rpf2-TAP, 90S/early 66S pre-ribosomes with Rrp5-TAP, middle 66S pre-ribosomes with Nsa1-TAP, and late 66S pre-ribosomes with Arx1-TAP (refer to Figure 2D). After Has1 depletion, few overall changes were observed in the protein constituents of consecutive intermediates, indicating that pre-ribosomes were largely intact (Supplementary Figure S3A). However, we did observe an increase in high molecular weight proteins in Nop7-, Rpf2- and Nsa1-TAP after Has1 depletion. These changes reflect a shift to earlier pre-ribosomal intermediates (compare with Rrp5-TAP), consistent with blocks in 35S and 27S pre-rRNA processing after Has1 depletion.

To more thoroughly determine how Has1 depletion affects protein association with 66S pre-ribosomes, we carried out iTRAQ mass spectrometry to compare the protein composition of Rpf2-TAP particles in the presence and absence of Has1. We carried out iTRAQ analysis after a 16 h shift to glucose-containing medium because this is the time point when Has1 was most depleted from 66S pre-ribosomes (Supplementary Figure S3B). Importantly, cells were still alive after this shift and were able to generate wild-type 66S pre-ribosomes after a 4 h shift back to galactose-containing medium to turn back on *HAS1* expression. Defects are qualitatively the same after a 4 h depletion as after a 16 h depletion (Supplementary Figure S3B, Figure 1C and D).

**Figure 4.**
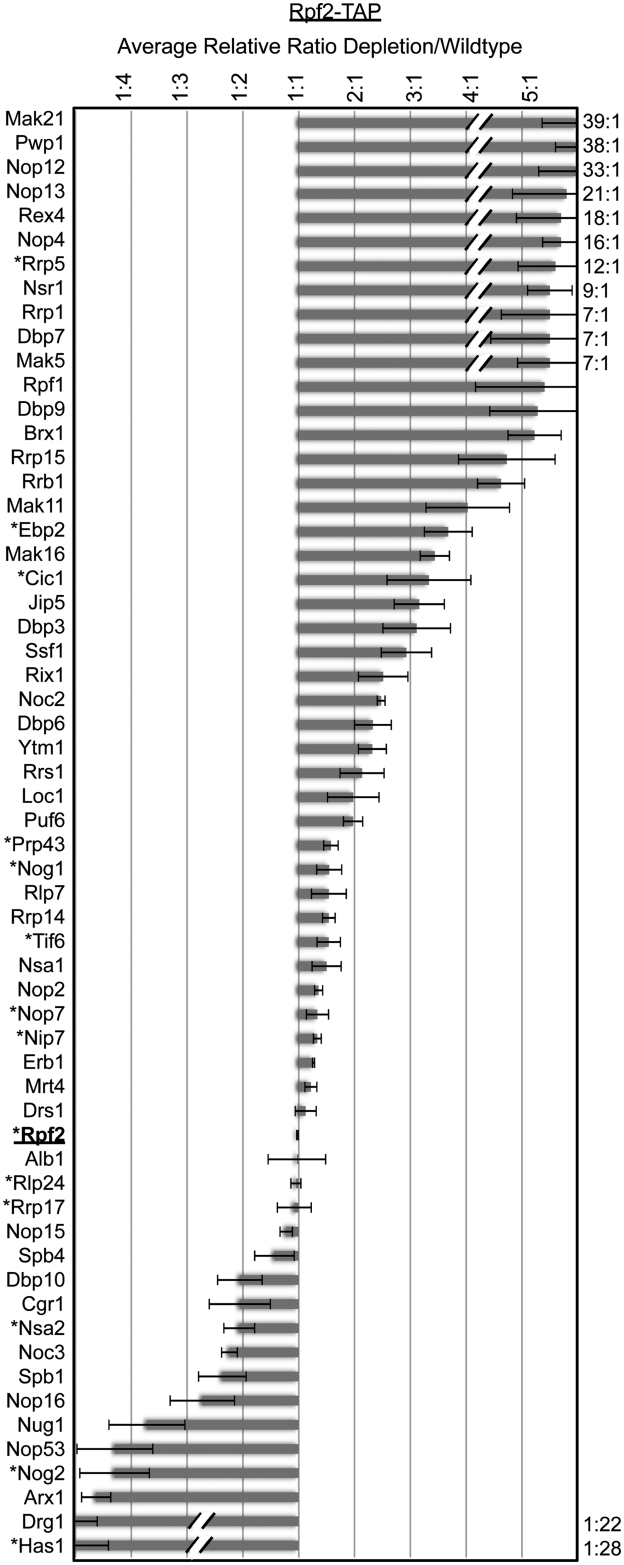
Has1 is required for maturation of early 66S pre-ribosomes. iTRAQ mass spectrometry was carried out to compare the average relative ratios of proteins found in Rpf2-TAP particles in the absence and presence of the Has1 protein (average relative ratio depletion/wild type). Fold changes in 66S assembly factor association are shown. Asterisks indicate that changes were verified by western blotting. Data represent the average of two technical replicates with error bars representing standard error of the mean.

The changes that we observed for 66S assembly factors after Has1 depletion largely correlate with their timing of association (Figure 4). For example, levels of early associating factors such as Dbp7 and Mak5 increased, whereas late associating factors such as Arx1 and Drg1 decreased (34). Therefore, many of the observed changes can be attributed to a relatively early block in maturation of 66S pre-rRNPs.

Consistent with western blot data (Figure 3), A_3_ factors (Nop7, Ytm1, Erb1, Rlp7, Cic1, Nop15) remained associated with pre-ribosomes in the absence of Has1 (Figure 4). Furthermore, Rrp17, one of the exonucleases that removes ITS1 from 27SA_3_ pre-rRNA, was still present in 66S particles. Although we were unable to detect Rat1, the other exonuclease involved in this step, it has previously been shown that Rat1 can enter pre-ribosomes in the absence of the A_3_ factors and Has1 (18). Furthermore, most of the factors required for C_2_ cleavage of 27SB pre-rRNA (Nop2, Nip7, Rpf2, Rrs1, Tif6, Rlp24, Nog1, Mak11) (33) were able enter 66S pre-ribosomes when Has1 was depleted, with the exceptions of Spb4, Dbp10, Nsa2 and Nog2. Levels of RNA helicases Sbp4 and Dbp10 decreased slightly, suggesting that Has1 may work together with these proteins to stabilize 27SB particles for Nog2 recruitment. Taken together, these data support a direct role of Has1 in triggering 27SA_3_ pre-rRNA processing and an indirect role in cleavage of the 27SB pre-rRNA.

### Has1 is necessary for stable association of r-proteins with domains I and III of 5.8S/25S rRNA

**Figure 5.**
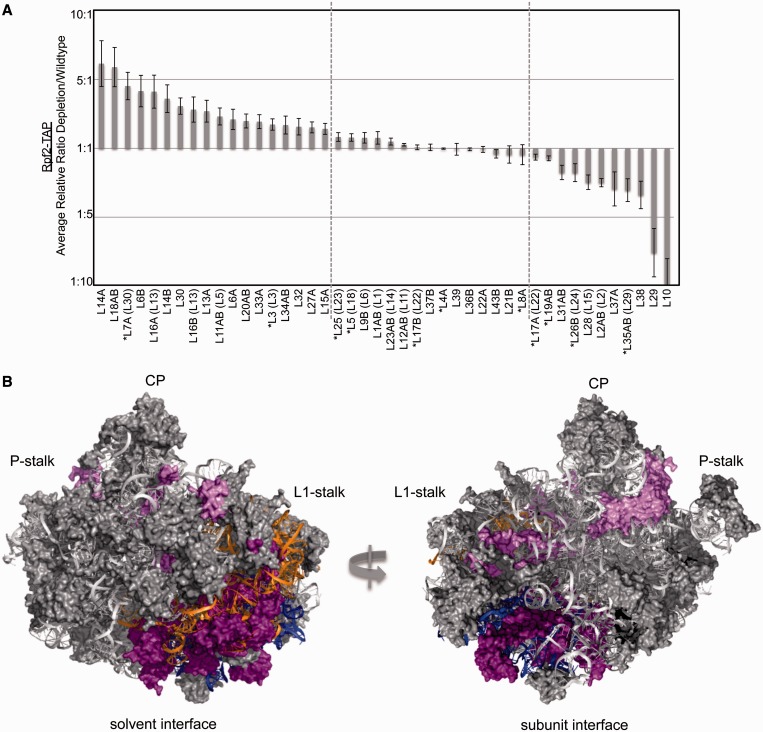
Has1 is required for r-protein association with domains I and III of 5.8S/25S rRNA. (**A**) Changes in large subunit r-protein association are shown as in Figure 4. Vertical dashed lines distinguish significant changes in protein association as a result of Has1 depletion. Proteins that increase are on the left, proteins that do not change are in the middle and proteins that decrease are on the right. Parentheses indicate bacterial homologues of yeast r-proteins. Asterisks indicate that changes were verified by western blotting. (**B**) PyMOL representations of large subunit r-proteins that change in the absence of Has1. R-proteins that remain associated with Rpf2-TAP after Has1 depletion are gray. Early associating r-proteins that are not stably associated with pre-ribosomes after Has1 depletion are dark pink (L17, L19, L26, L31, L35, L37, L38) and late associating r-proteins that are not stably associated are light pink (L2, L10, L28, L29). R-proteins that were not detected by mass spectrometry are not shown. The location of rRNA domains I and III are indicated in orange and blue, respectively, to highlight clustering of proteins that decrease association with 66S pre-ribosomes in the absence of Has1. Locations of the central protuberance (CP), P-stalk and L1-stalk are indicated.

We observed changes in association of large subunit r-proteins with Rpf2-TAP particles that correlate with the order in which these proteins function in 66S subunit maturation (31,35). For example, levels of r-proteins required for early processing steps, including L3, L7, L8, L16, L18, L20, L32 and L33, increased in Rpf2-TAP particles in the absence of Has1, whereas levels of r-proteins required for late processing steps such as L2, L10, L28 and L29 decreased (Figure 5A). Furthermore, because L29 and L10 do not join pre-ribosomes and L28 and L2 are not stably associated with pre-ribosomes until after the 27SB pre-rRNA processing step is complete, the absence of these r-proteins likely reflects the block in pre-rRNA processing after Has1 depletion (Figure 5B, light purple) [P. Milkereit, manuscript submitted, (36)]. Notably, most of the early associating large subunit r-proteins that decreased association with pre-ribosomes in the absence of Has1 bind to domain I where 5.8S rRNA base pairs with 25S rRNA (L17, L26, L35, L37) and domain III of 25S rRNA (L19, L31, L38) (Figure 5B, dark purple), suggesting that Has1 may be involved in stabilizing these domains of the 60S subunit.

### Has1 stabilizes domain I of 5.8S/25S rRNA

To determine the role of Has1 in domain I folding and r-protein binding, we carried out *in vivo* DMS structure probing followed by primer extension of modified RNA. To specifically determine how the structure of domain I changes within pre-ribosomes and not mature rRNA, we used primers that bind within ITS2 for primer extension analysis. DMS modifies adenines and cytosines that are not protected by protein or involved in Watson–Crick base pairing. Therefore, by comparing the DMS modification pattern of 5.8S rRNA in the presence and absence of Has1, we could identify changes in r-protein binding to domain I, base pairing within 5.8S rRNA and base pairing between 5.8S and 25S rRNAs.

**Figure 6.**
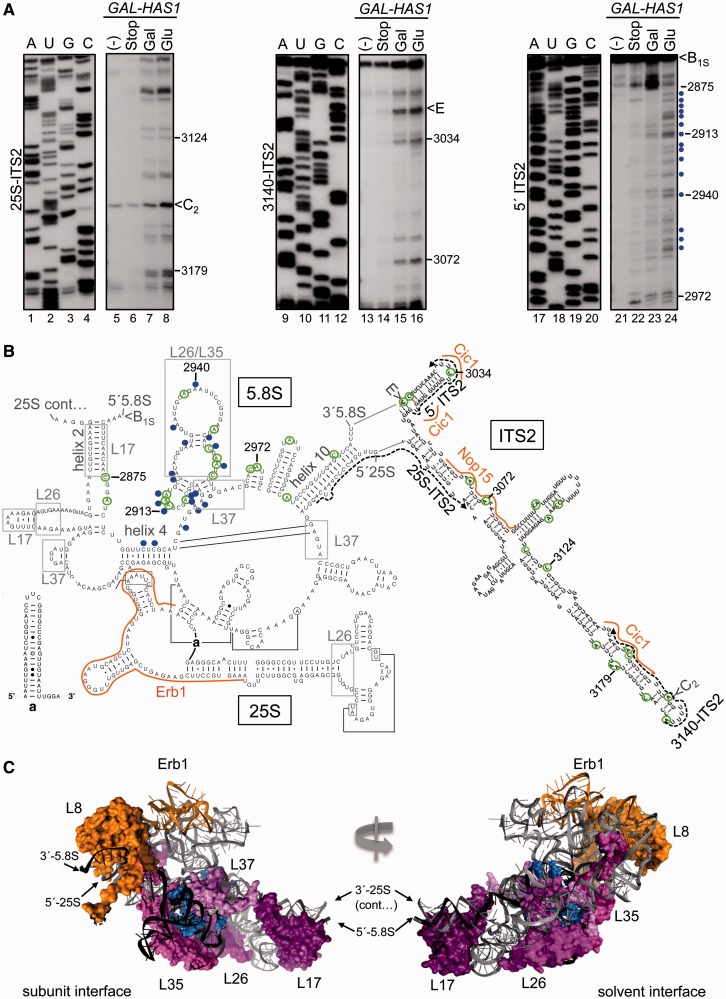
Sequences in 5.8S rRNA are more modified by DMS in the absence of Has1. (**A**) *In vivo* DMS probing of the *GAL-HAS1* strain grown in galactose- (Gal) or glucose- (Glu) containing medium. Controls include no DMS control (−) and stop control (Stop), in which β-mercaptoethanol was added to quench the reaction before addition of DMS. Control reactions were carried out on RNA that was isolated from the *GAL-HAS1* strain grown in galactose-containing medium. For comparison, the no DMS control was also carried out on RNA that was extracted from the *GAL-HAS1* strain grown in glucose (Supplementary Figure S4B). The corresponding sequencing ladders are shown (lanes A,U,G,C). The 25S-ITS2, 3140-ITS2 and 5′ ITS2 primers were used for primer extension of extracted RNA. Nucleotides that become more modified in the absence of Has1 are indicated by blue circles. Nucleotide positions are indicated for 35S pre-rRNA. The same RNA samples and loading were used for all three sets of primer extensions. For better separation of primer extension products ending at 2875–2913 nt refer to Supplementary Figure S4A. (**B**) Locations of nucleotides that are more modified in the absence of Has1 (blue circles) are displayed on the 5.8S/25S rRNA secondary structure (domain I) (http://www.rna.ccbb.utexas.edu/) and the predicted hairpin structure of ITS2 (53). The chemical modification pattern in wild-type cells (green circles) corresponds to the predicted secondary structure of domain I and ITS2. Locations of primers used for primer extension are shown as dashed arrows. Orange lines represent the binding sites of Erb1, Nop15 and Cic1 determined by cross-linking and analysis of cDNAs (39). Helices where 5.8S rRNA base pairs with 25S rRNA (helix 2, 4, 10) and binding sites of r-proteins (gray boxes) are indicated. (**C**) PyMOL representation of domain I of the 5.8S/25S rRNA of the 60S subunit (5.8S rRNA black, 25S rRNA gray). R-protein L8 and the RNA binding site of Erb1 are orange. R-proteins that decrease association with 66S pre-ribosomes in the absence of Has1 (L17, L26, L35, L37) are shades of pink and purple. Nucleotides that are more modified in the absence of Has1 are blue spheres.

In the absence of Has1, we observed significant changes in the chemical modification pattern of 5.8S rRNA (Figure 6A, lanes 23 and 24 and Supplementary Figure S4A, lanes 7 and 8), but not ITS2 (Figure 6A, lanes 7 and 8 and 15 and 16). Residues that were more modified by DMS in the absence of Has1 cluster to a region of 5.8S rRNA between helix 4 and helix 10 (Figure 6B, blue circles) that corresponds to the binding sites of L26, L35 and L37 (gray boxes) (25,32). Additional changes were observed in helix 4 where 5.8S rRNA base pairs with 25S rRNA, suggesting that intermolecular base pairing is compromised in the absence of Has1. On the other hand, the secondary structure of the ITS2-proximal helix 10 and ITS2 appear to be intact. We also assayed for rearrangements in ITS1 and the 3′ end of 18S rRNA using ITS1-specific primers, but did not identify any noticeable changes in these regions after depletion of Has1. Thus, Has1 is required for stable incorporation of r-proteins into domain I of 5.8S/25S rRNA and may help facilitate base pairing of helix 4 between 5.8S and 25S rRNAs.

### Association of Has1 with 66S pre-ribosomes is ATP-independent

Next, we dissected the function of conserved ATP binding and helicase motifs of Has1 in ribosome biogenesis. We generated several *has1* mutant strains in which expression of the HA-tagged genomic wild-type *HAS1* allele was driven by the *GAL* promoter, and an untagged *has1* mutant allele was controlled by its endogenous promoter on a plasmid. Therefore, when these strains were shifted to glucose-containing medium, only the *has1* mutant allele was expressed, allowing us to assay defects associated with potentially inviable *has1* mutants.

**Figure 7.**
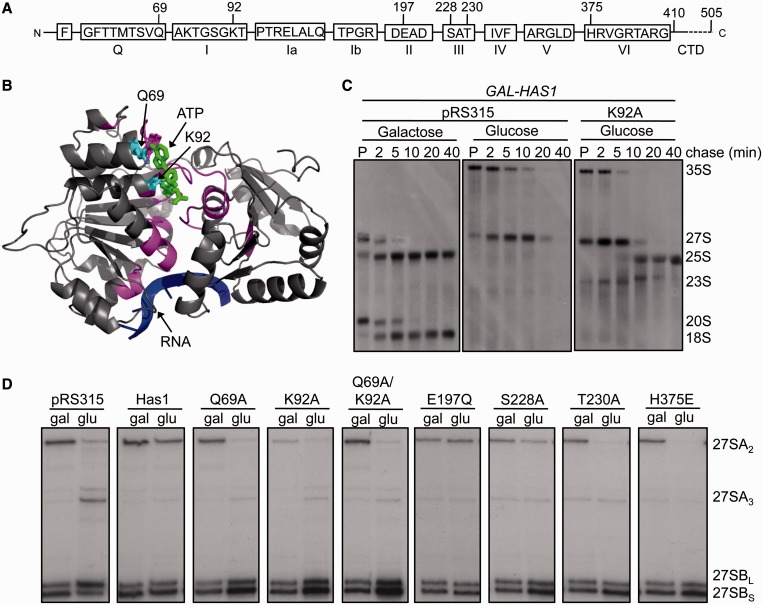
Systematic analysis of *has1* mutants reveals distinct ATP-independent and ATP-dependent roles of Has1 in 27S pre-rRNA processing. (**A**) Conserved DEAD-box motifs of Has1 are shown with mutated residues indicated. (**B**) Pymol representation of the predicted structure of Has1 bound to RNA (blue) and ATP (green) (see ‘Materials and Methods’ section). Amino acids Q69 and K92A (cyan) of motifs Q and I, respectively, are predicted to contact ATP. Conserved helicase domains are pink. The C-terminal domain (CTD) was not modeled on this structure. (**C**) Pulse-chase analysis of *has1* mutants. *GAL-HAS1* strains carrying *LEU2-*marked plasmids containing no insert (pRS315) or the K92A *has1* mutant allele were grown in galactose- or glucose-containing medium lacking methionine and leucine. Pulse-chase analysis was carried out as in Figure 1B. The positions of RNAs are indicated (right). Data for the K92A mutant strain grown in galactose-containing media are not shown because it was identical to the pRS315 strain. (**D**) *GAL-HAS1* strains carrying plasmids containing no insert (pRS315), wild-type *HAS1* or *has1* mutant alleles (top) were grown in galactose- (gal) or glucose- (glu) containing medium followed by RNA extraction and primer extension with oligo f as in Figure 1D. RNA intermediates are indicated (right).

Mutations were generated in conserved motifs Q (Q69A), I (K92A), II (E197Q), III (S228A, T230A) and VI (H375E) (Figure 7A). *In vivo*, overexpression of the K92A mutant protein has a dominant negative effect on growth (37). *In vitro*, the K92A mutant protein lacks ATPase and helicase activity and has 20-fold reduced affinity for ATP, the S228A and T230A mutant proteins have reduced ATPase and helicase activity and the H375E mutant protein retains ATPase activity but lacks helicase activity (16). The Q motif is involved in ATP binding (38). Therefore, the Q69A mutation would likely result in decreased ATP binding. We predict that alanine substitutions of both Q69 and K92 would abolish ATP binding by Has1 (Figure 7B). We also made a mutation in motif II (E197Q) predicted to decrease ATPase activity of DEAD-box proteins (6).

The Q69A, K92A, Q69A/K92A, T230A and H375E mutants were inviable, indicating that ATP-dependent activities of Has1 are essential (Supplementary Figure S5A). The E197Q and S228A mutants were viable, indicating that these residues are not essential for Has1 function *in vivo*. The S228A mutant protein retained most ATPase and helicase activity *in vitro* and caused cold sensitivity *in vivo* (16).

**Figure 8.**
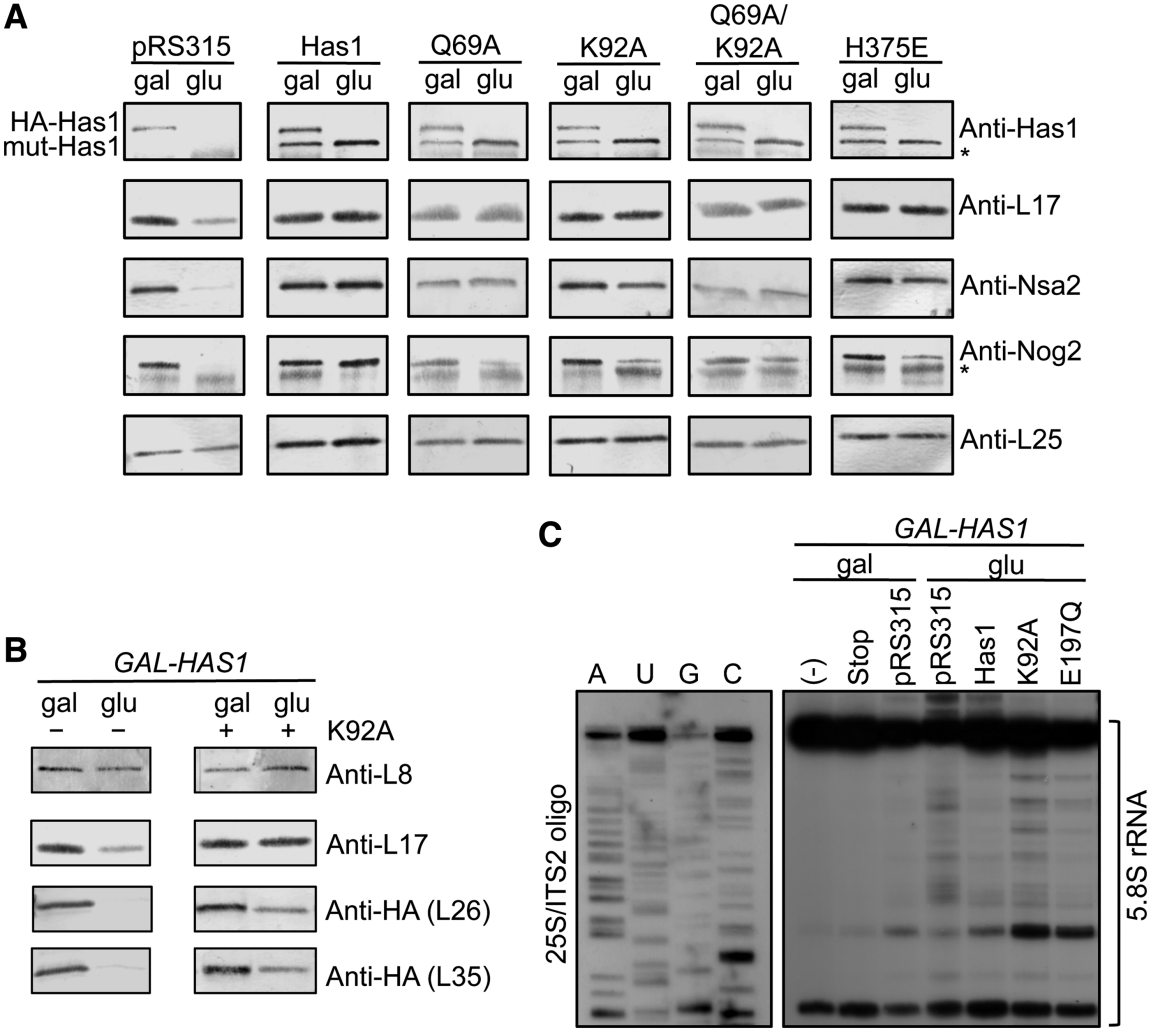
ATP binding by Has1 is required for stable association of r-proteins L26 and L35 with pre-ribosomes, but not L17. (**A**) Rpf2-TAP was used to purify pre-ribosomes from strains grown as in Figure 7D (top), and associated proteins were assayed by western blotting (right). For anti-Has1 panels, the top band corresponds to the HA-tagged wild-type copy of Has1 (HA-Has1) and the bottom band corresponds to the untagged mutant version of Has1 (mut-Has1). Asterisks indicate IgG stripped from beads during purification. L25, loading control. (**B**) *GAL-HAS1* strains carrying an empty vector control (−) or K92A mutant allele (+) were grown in galactose- (gal) or glucose- (glu) containing medium followed by pre-ribosome purification using Rpf2-TAP. Associated proteins were assayed by western blotting (right). L8, loading control. (**C**) *In vivo* DMS probing of *has1* mutants. DMS probing was carried out as in Figure 6, except that *GAL-HAS1* strains carrying the pRS315 plasmid backbone or plasmids containing wild-type *HAS1* or mutant *has1* alleles were used. The 25S/ITS2 oligo, which selects for 27S pre-rRNA intermediates, was used for primer extension analysis.

All of the mutant Has1 proteins were able to enter Rpf2-TAP containing pre-ribosomes (Figure 8A), indicating that ATP binding, as well as ATPase and helicase activity of Has1 are dispensable for association with 66S pre-ribosomes. These ATP-dependent activities of Has1 are also not required for association with Rrp5-TAP, which includes 90S/early 66S pre-ribosomes (Supplementary Figure S5B). Furthermore, the K92A and H375E mutant proteins were not found associated with Arx1-TAP, suggesting that the lack of ATP binding and helicase activity does not prevent or delay exit of Has1 from 66S pre-ribosomes (Supplementary Figure S5B). In wild-type cells, Has1 exits 66S pre-ribosomes before Arx1 entry (Figure 2D).

### Has1 carries out ATP-independent and ATP-dependent roles in 66S pre-ribosome maturation

We next examined the levels of 18S and 25S rRNAs in the mutants to estimate the effects of individual mutations on 40S and 60S ribosomal subunit production (Supplementary Figure S5C). Mutations predicted to abolish or reduce ATP binding or helicase activity caused a reduction in 18S rRNA with little or no effect on 25S rRNA (lanes Q69A, K92A, Q69A/K92A, T230A, H375E). The severity of these defects correlated with the ability of the mutant protein to support growth in the absence of wild-type Has1 (Supplementary Figure S5A). Therefore, ATPase and/or helicase activities of Has1 are essential for 18S rRNA production.

To determine the role of Has1 ATP-dependent activity in pre-rRNA processing and turnover, we carried out pulse-chase analysis of nascent pre-rRNAs in the presence of wild-type Has1, no Has1 or the K92A mutant protein. When Has1 was expressed, 27S pre-rRNA was almost completely processed to 25S rRNA after 5 min (Figure 7C, lanes pRS315, galactose). However, when Has1 was depleted, 27S pre-rRNA processing was delayed followed by turnover after 10 min (lanes pRS315, glucose, see also Figure 1B). When the K92A mutant protein was the only version of Has1 expressed, processing of 27S pre-rRNA to 25S rRNA was significantly delayed (compare lanes K92A, glucose with pRS315, galactose). However, stalled intermediates were not considerably turned over. Consistent with reduced steady-state levels of 18S rRNA detected in *has1* mutants (Supplementary Figure S5C), the K92A mutant did not make 20S pre-rRNA or 18S rRNA (Figure 7C, lanes K92A). Therefore, ATP binding by Has1 is required for 18S rRNA production and efficient 27S pre-rRNA processing.

We next examined the effects of the individual mutations on steady-state levels of 27S pre-rRNA intermediates. The processing defects associated with Has1 depletion (Figure 7D, lanes pRS315) were consistent with earlier observations (Figure 1D). These include a defect in 27SA_3_ pre-rRNA processing as indicated by an increase in 27SA_3_ and decrease in 27SB_S_ pre-rRNA, as well as a defect in 27SB pre-rRNA processing as indicated by an increase in 27SB_L_ pre-rRNA. Furthermore, consistent with functions in A_0_, A_1_ and A_2_ cleavages of 35S pre-rRNA, levels of 27SA_2_ pre-rRNA were reduced. On the other hand, when Has1 was able to associate with pre-ribosomes but did not retain ATP binding activity, there was primarily a defect in C_2_ cleavage as indicated by an increase in 27SB_L_ and 27SB_S_ pre-rRNAs (Figure 7D, lanes Q69A, K92A, Q69A/K92A). Mutant proteins expected to retain ATP binding with reduced helicase activity showed milder effects on 27SB pre-rRNA processing (lanes S228A, T230A, H375E). Furthermore, the E197Q mutation, which had no effect on growth, did not affect 27SB pre-rRNA processing (lanes E197Q). In contrast to depletion of Has1, in which 7S pre-rRNA levels decreased, Has1 missense mutations resulted in increased levels of 7S pre-rRNA, with no effect on (Supplementary Figure S5D, lanes S228A, T230A, H375E) or decreased levels of (lanes Q69A, K92A, Q69A/K92A) 5.8S rRNA. This is consistent with pulse chase data showing delayed pre-rRNA processing in the K92A mutant strain. Therefore, ATP-dependent activity of Has1 is required for efficient 27SB and 7S pre-rRNA processing.

We also assayed for changes in protein association with Rpf2-TAP particles in each mutant (Figure 8A and B). We were able to separate the effects on pre-ribosomal protein recruitment into two categories, proteins that required the presence of Has1 in pre-ribosomes for their assembly with pre-rRNPs (L17, Nsa2) and proteins that more specifically required Has1 ATP-dependent activities for stable association (L26, L35, Nog2). Furthermore, the chemical modification pattern of 5.8S rRNA in the absence of ATP binding was consistent with decreased association of L26, L35 and L37 with pre-ribosomes (Figure 8C, lane K92A). Taken together, these experiments demonstrate that Has1 is a multifunctional protein that plays separate scaffolding and enzymatic roles in consecutive 66S pre-rRNP maturation steps.

## DISCUSSION

Here, we demonstrate that the essential, conserved DEAD-box protein Has1 plays a *bona fide* role in biogenesis of 60S ribosomal subunits in yeast. We reveal ATP-independent and ATP-dependent functions of Has1 in proper formation of an rRNP neighborhood within assembling ribosomes and provide evidence for coordination of rRNP conformational changes with consecutive pre-rRNA processing steps. Furthermore, in the absence of Has1, aberrant intermediates are targeted for irreversible turnover. Taken together, this work illustrates the role of Has1 in 66S pre-rRNP maturation and processing and provides a framework to understand the functions of other DEAD-box proteins in ribosome assembly.

### Model for Has1 recruitment to 66S pre-ribosomes

**Figure 9.**
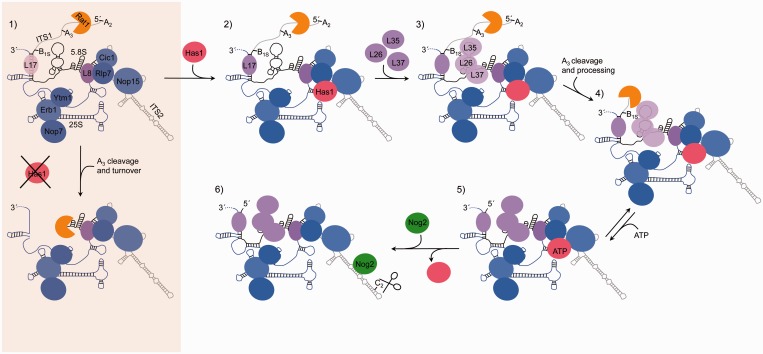
Model for Has1 recruitment and function during 66S pre-rRNP maturation. Maturation of 27S pre-rRNAs with respect to Has1 function is shown (steps 1–6). In the absence of Has1, 27S pre-rRNA is turned over (shaded box). Sequences of 25S rRNA are blue, 5.8S rRNA are black and ITS1 and ITS2 are gray. See ‘Discussion’ section for detailed explanation of model. The binding sites of Has1 and Nog2 are not known.

Our data support a model in which Has1 is recruited to 66S pre-ribosomes by Rlp7 or Nop15 and/or by interactions with RNA structures that are stabilized by the A_3_ factors (Figure 9, steps 1–2). Before Has1 binding, r-protein L8 and A_3_ factors Nop7, Ytm1, Erb1, Nop15, Cic1 and Rlp7 associate with 35S or 27SA_2_ pre-rRNAs (step 1) (18,31). Has1 depends on these proteins to enter 66S pre-ribosomes (Figure 3A and B) and interacts with Rlp7 and Nop15 in two-hybrid assays (Figure 3D). Nop15 binds to sequences in ITS2 (39), and Rlp7 binds to the ITS2 proximal helix 10 (unpublished observations, J. de la Cruz and M. Fromont-Racine, personal communication). Thus, recruitment by Nop15 or Rlp7 would place Has1 in close proximity to its place of action to regulate conformational changes within domain I of 5.8S/25S rRNA.

### Coupling domain I assembly with 27SA_3_ pre-rRNA processing

Has1 is the last known factor in the assembly hierarchy to trigger processing of 27SA_3_ pre-rRNA and drive stable incorporation of r-proteins L17, L26, L35 and L37 with domain I (Figure 3, illustrated in Figure 9, steps 1–4). A previous study demonstrated that the A_3_ factors are required for 27SA_3_ pre-rRNA processing and r-protein association (18). In the absence of these A_3_ factors, Has1 was not able to associate with pre-ribosomes. Here, we demonstrate that all other proteins required for 27SA_3_ pre-rRNA processing and r-protein binding to domain I are present in pre-ribosomes after Has1 depletion (Figure 4), supporting a direct role of Has1 in these steps.

A likely scenario is that Has1 binding plays a role in RNP rearrangements that couple 27SA_3_ pre-rRNA processing with stable association of r-proteins with domain I. In support of this, exonucleases Rat1 and Rrp17, which process 27SA_3_ pre-rRNA, remain bound to pre-ribosomes in the absence of Has1 [Figure 4 and (18)] but cannot carry out pre-rRNA processing. One possibility is that Has1 binding disrupts an alternative pre-rRNA conformation that inhibits exonucleolytic processing and binding of r-proteins with rRNA. Sequences in ITS1 have the potential to base pair with 5.8S rRNA in a conformation that would preclude formation of helix 2, partially destabilize helix 4 and inhibit removal of ITS1 (40,41). Destabilization of this alternative structure by Has1 would enable base pairing between 5.8S and 25S rRNAs, binding of L17 with rRNA, and efficient processing of 27SA_3_ pre-rRNA.

Another possibility is that Has1 binding drives removal of early associating assembly factors that prevent r-protein binding to domain I and 27SA_3_ pre-rRNA processing. In support of this, Rrp5, Nop12, Nop4, Mak21/Noc1 and Pwp1 levels within Rpf2-TAP particles increase in the absence of Has1 (Figure 4). Rrp5 binds to sequences on either side of the A_3_ site and may physically block exonucleolytic processing (42). Nop12 cross links to 5.8S rRNA adjacent to the L26, L35 and L37 binding sites, and Nop4 cross-links to domain III of 25S rRNA where it could potentially inhibit stable association of r-proteins with domain I (39). Furthermore, Mak21/Noc1 forms a subcomplex with Rrp5 (43) and Pwp1 interacts with Nop12 (44). These proteins may hold the pre-ribosome in an immature state that would prevent stable r-protein binding to domain I and pre-rRNA processing prior to Has1 association.

### Aberrant pre-ribosomes are turned over in the absence of Has1

In the absence of Has1, 27S pre-rRNA processing is delayed followed by turnover of aberrant pre-rRNAs (Figure 1B). One possibility is that the absence of r-proteins associated with domains I and III exposes RNA to endo- and exonucleases allowing pre-ribosome to be targeted for turnover (Supplementary Figure S6A compared with Supplementary Figure S6C). In support of this, a previous study demonstrated that when A_3_ factors are absent from pre-ribosomes, the processing exonuclease Rat1 participates in turnover of abortive intermediates (18). Furthermore, the absence of r-protein L8 results in a more significant decrease in association of r-proteins with domains I and III than after Has1 depletion (Supplementary Figure S6B), which correlates with more rapid turnover of 27S pre-rRNAs (31). The rate of turnover observed is much less after depletion of Has1 than after depletion of L8 but more than that observed after depletion of individual r-proteins L17, L35 and L37 (32). Thus, there is a direct correlation between the extent of r-protein binding to domain I and 27S pre-rRNA turnover. Furthermore, r-protein L17 functions as a roadblock to halt 27SA_3_ pre-rRNA processing precisely at the B_1S_ site in wild-type cells (18). Therefore, after Has1 depletion, the absence of L17 and destabilization of domain I may allow 27S pre-rRNA turnover (Figure 9, shaded box).

### ATP binding by Has1 promotes efficient assembly and processing of 66S pre-rRNPs

An interesting observation from this study is that conserved motifs required for ATP binding by Has1 are not required for Has1 function in 27SA_3_ pre-rRNA processing, but appear to drive efficient stabilization of domain I and are required for downstream pre-rRNA processing steps (Figure 9, steps 4–5). In the absence of ATP-dependent activity of Has1, the levels of L26 and L35 in 66S pre-ribosomes are reduced (Figure 8B). Furthermore, the nucleotides that are normally protected from chemical modification by L26, L35 and L37 are more accessible to chemical modification (Figure 8C). Pulse-chase and steady-state analyses demonstrate that mutations predicted to abolish ATP binding by Has1 result in delayed 27S pre-rRNA processing to 25S rRNA and reduced levels of 5.8S rRNA (Figure 7C, Supplementary Figure S5D). Therefore, Has1 enzymatic activity is required for optimal 66S pre-ribosome maturation.

One possibility is that ATP-independent binding of Has1 to RNA initially lowers the free energy landscape to promote correct folding of domain I. By coupling RNA binding to ATP binding and hydrolysis, Has1 may be able to further overcome energetic barriers and propagate the formation of the native state of domain I (45). Therefore, in the absence of ATP-dependent activity of Has1, these structural transitions may be less energetically favorable and occur more slowly. Consistent with this hypothesis, processing of 27S pre-rRNAs to mature 25S rRNA takes at least twice as long for the K92A mutant as wild-type cells (Figure 7C).

There are several examples of RNA helicases that exhibit ATP-independent strand annealing and RNA unwinding activities (46–50). For example, the DEAD-box protein Rok1 has been shown to facilitate ATP-independent strand annealing of a pre-rRNA structure (50). Furthermore, Prp22 has ATP-independent functions in the second step of pre-mRNA splicing and an ATP-dependent role in spliceosome disassembly (47). Here, coupling ATP-independent and ATP-dependent functions may allow Has1 to maintain the proper order of consecutive pre-rRNA processing steps.

### How does Has1 exit pre-ribosomes?

After formation of the 27SB pre-rRNA, Has1 exits pre-ribosomes (Figure 9, steps 5–6), which might allow Nog2 to associate and trigger downstream cleavage and processing of ITS2 (Figure 9, step 6). The timing of Has1 release from pre-ribosomes coincides with Nog2 entry (Figure 2C and D), and these two proteins are mutually exclusive for association with 27SB pre-rRNAs (Supplementary Figure S2). It is also possible that Nog2 GTPase activity drives release of Has1 from pre-ribosomes, but that this occurs so rapidly that we cannot detect both proteins in the same pre-rRNP.

### Communication between 40S and 60S subunit biogenesis

There is evidence for cross talk between the 40S and 60S subunit biogenesis pathways, but molecular details of this communication are not understood. Has1 is one of three assembly factors known to function in both 43S and 66S particle maturation, including the RNA-binding protein Rrp5 (9) and DEAD-box protein Prp43 (10). Because Has1 is able to associate with both 43S and 66S pre-ribosomes (Figure 2), we suspect that it has multiple binding sites on the pre-rRNA to facilitate separate functions in 40S and 60S subunit biogenesis, similar to Prp43 (51). However, we have not been able to identify the binding sites of Has1 on pre-rRNA using cross-linking techniques (unpublished observations). This could potentially be because Has1 contacts the phosphodiester backbone of RNA, not allowing efficient cross-linking to nucleotide bases. In the future, it would be of interest to more carefully dissect the function of Has1 in 40S subunit assembly.

To our knowledge, this is the most thorough examination of the protein composition and RNA structure of pre-ribosomes in the absence of a DEAD-box protein. Previously, DEAD-box proteins have been suggested to function in snoRNA release from pre-ribosomes (13,52). This study highlights the importance of a DEAD-box protein in pre-rRNP maturation, including facilitating pre-rRNA folding and processing, as well as binding of r-proteins and assembly factors.

## SUPPLEMENTARY DATA

Supplementary Data are available at NAR Online: Supplementary Tables 1 and 2, Supplementary Figures 1–6, Supplementary Methods and Supplementary References [54–56].

## FUNDING

National Institutes of Health (NIH) [R01-GM028301 to J.L.W., F32-GM099225 to J.A.D.]. Funding for open access charge: NIH.

*Conflict of interest statement*. None declared.

## Supplementary Material

Supplementary Data
