# Supplementary material for: Has1 regulates consecutive maturation and processing steps for assembly of 60S ribosomal subunits
Source: Nucleic Acids Res. 2013 Jun 20;41(16):7889–904. doi: 10.1093/nar/gkt545 (PMC3763536; doi:10.1093/nar/gkt545)
Supplement: Supplementary Data [file supp_41_16_7889__index.html]

Has1 regulates consecutive maturation and processing steps for assembly of 60S ribosomal subunits — Has1 regulates consecutive maturation and processing steps for assembly of 60S ribosomal subunits — Supplementary Data 

# Has1 regulates consecutive maturation and processing steps for assembly of 60S ribosomal subunits

## 

files

**Files in this Data Supplement:**

- Supplementary Data - pdf file
- Supplementary Data - xlsx file
