# Supplementary material for: Has1 regulates consecutive maturation and processing steps for assembly of 60S ribosomal subunits
Source: Nucleic Acids Res. 2013 Jun 20;41(16):7889–904. doi: 10.1093/nar/gkt545 (PMC3763536; doi:10.1093/nar/gkt545)
Supplement: Supplementary Data [file supp_gkt545_nar-01122-r-2013-File011.pdf]

SUPPLEMENTARY DATA

Has1 regulates consecutive maturation and processing steps for assembly of 60S ribosomal subunits

Jill A Dembowski, Benjamin Kuo and John L Woolford Jr

Table SI. Yeast strains used in this study.

Strain	Genotype	Source
BY4741	*MATa his3Δ1 leu2Δ0 ura3Δ0 met15Δ0*	Euroscarf
JWY6147	*MATa ura3-52 trp1-Δ101 lys2-801 his3-Δ200 leu2-Δ1*	Dr. Elizabeth Jones
JDY10-3B (pAS24-HAS1)	*MATa ade2-1 his3-11 leu2-3,112 ura3-1 can1-100 has1::HIS3* [pAS24-*HAS1*]	(8)
JWY9299	*MATa ura3-52 trp1-Δ101 lys2-801 his3-Δ200 leu2-Δ1 has1::GAL-3HA-HAS1 HIS3*	This study
JWY8243	*MATa ura3-52 trp1-Δ101 lys2-801 his3-Δ200 leu2-Δ1 nop7::GAL-3HA-NOP7 TRP1*	(18)
JWY6851	*Matα his3-Δ200 trp1-Δ101 leu2-Δ2 ura3-167 rps14a::KANMX6 rps14b::LEU2* [*pGAL-RPS14A*]	(54)
JWY9261	*MATa his3Δ1 leu2Δ0 ura3Δ0 met15Δ0 has1::HAS1-HTP URA3*	This study
JWY8200	*MATa ura3-52 trp1-Δ101 lys2-801 his3-Δ200 leu2-Δ1 nop7::NOP7-TAP URA3*	(33)
JWY8308	*MATa ura3-52 trp1-Δ101 lys2-801 his3-Δ200 leu2-Δ1 rpf2::RPF2-TAP URA3*	(18)
JWY7767	*MATa his3Δ1 leu2Δ0 met15Δ0 ura3Δ0 enp1::ENP1-TAP HIS3*	Open Biosystems
JWY8826	*MATa his3Δ1 leu2Δ0 met15Δ0 ura3Δ0 rrp5::RRP5-TAP HIS3*	Open Biosystems
JWY8814	*MATa his3Δ1 leu2Δ0 met15Δ0 ura3Δ0 nsa1::NSA1-TAP HIS3*	Open Biosystems
JWY8809	*MATa his3Δ1 leu2Δ0 met15Δ0 ura3Δ0 nog2::NOG2-TAP HIS3*	Open Biosystems
JWY8808	*MATa his3Δ1 leu2Δ0 met15Δ0 ura3Δ0 rix1::RIX1-TAP HIS3*	Open Biosystems
JWY8811	*MATa his3Δ1 leu2Δ0 met15Δ0 ura3Δ0 arx1::ARX1-TAP HIS3*	Open Biosystems
JWY7769	*MATa his3Δ1 leu2Δ0 met15Δ0 ura3Δ0 nob1::NOB1-TAP HIS3*	Open Biosystems
JWY8591	*MATa ura3-52 trp1-Δ101 lys2-801 his3-Δ200 leu2-Δ1 rpf2::RPF2-TAP URA3 rpl8a::KANMX6 rpl8b::GAL-3HA-RPL8B HIS3*	(31)
JWY8271	*MATa ura3-52 trp1-Δ101 lys2-801 his3-Δ200 leu2-Δ1 nop7::GAL-3HA-NOP7 TRP1 rpf2::RPF2-TAP URA3*	(18)
JWY9622	*MATa ura3-52 trp1-Δ101 lys2-801 his3-Δ200 leu2-Δ1 rpl17b::KANMX6 rpl17a::GAL-3HA-RPL17A TRP1 nop7::NOP7-TAP URA3*	(32)
JWY8145	*MATα trp1 lys2-801 ura3-52 his3Δ200 pep4::HIS3 prb1Δ1.6R can nog2:GAL-3HA-NOG2 TRP1 rpf2:RPF2-3HA KANMX6 nop7:NOP7-TAP URA3*	(33)
PJ69-4a	*MATa trp1-901 leu2-3,112 ura3-52 his3-200 gal4Δ gal80Δ LYS2::GAL1-HIS3 GAL2-ADE2 met2::GAL7-lacZ*	(24)
PJ69-4α	*MATα trp1-901 leu2-3,112 ura3-52 his3-200 gal4Δ gal80Δ LYS2::GAL1-HIS3 GAL2-ADE2 met2::GAL7-lacZ*	(55)
JWY9309	*MATa ura3-52 trp1-Δ101 lys2-801 his3-Δ200 leu2-Δ1 has1::GAL-3HA-HAS1 HIS3 rpf2::RPF2-TAP URA3*	This study

JWY9314	*MATa ura3-52 trp1-Δ101 lys2-801 his3-Δ200 leu2-Δ1 has1::GAL-3HA-HAS1 HIS3 nop7::NOP7-TAP URA3*	This study
JWY9303	*MATa ura3-52 trp1-Δ101 lys2-801 his3-Δ200 leu2-Δ1 has1::GAL-3HA-HAS1 HIS3 rrp5::RRP5-TAP URA3*	This study
JWY9306	*MATa ura3-52 trp1-Δ101 lys2-801 his3-Δ200 leu2-Δ1 has1::GAL-3HA-HAS1 HIS3 nsa1::NSA1-TAP URA3*	This study
JWY9311	*MATa ura3-52 trp1-Δ101 lys2-801 his3-Δ200 leu2-Δ1 has1::GAL-3HA-HAS1 HIS3 arx1::ARX1-TAP URA3*	This study
JWY9318	*MATa ura3-52 trp1-Δ101 lys2-801 his3-Δ200 leu2-Δ1 has1::GAL-H3A-HAS1 HIS3 rpf2::RPF2-TAP URA3 rrp17::RRP17-3HA TRP1*	This study
JWY9342	*MATa ura3-52 trp1-Δ101 lys2-801 his3-Δ200 leu2-Δ1 has1::GAL-3HA-HAS1 HIS3 rpf2::RPF2-TAP URA3 rrp5::RRP5-3HA TRP1*	This study
JWY9338	*MATa ura3-52 trp1-Δ101 lys2-801 his3-Δ200 leu2-Δ1 has1::GAL-3HA-HAS1 HIS3 rpf2::RPF2-TAP URA3 rpl19A::RPL19A-3HA TRP1*	This study
JWY9340	*MATa ura3-52 trp1-Δ101 lys2-801 his3-Δ200 leu2-Δ1 has1::GAL-3HA-HAS1 HIS3 rpf2::RPF2-TAP URA3 rpl26A::RPL26A-3HA TRP1*	This study
JWY9341	*MATa ura3-52 trp1-Δ101 lys2-801 his3-Δ200 leu2-Δ1 has1::GAL-3HA-HAS1 HIS3 rpf2::RPF2-TAP URA3 rpl35B::RPL35B-3HA TRP1*	This study
JWY8275	*MATa ura3-52 trp1-Δ101 lys2-801 his3-Δ200 leu2-Δ1 rlp7::GAL-3HA-RLP7 TRP1*	(18)
JWY9327	*MATa ura3-52 trp1-Δ101 lys2-801 his3-Δ200 leu2-Δ1 has1::GAL-3HA-HAS1 HIS3 rpf2::RPF2-TAP URA3* [pRS315]	This study
JWY9328	*MATa ura3-52 trp1-Δ101 lys2-801 his3-Δ200 leu2-Δ1 has1::GAL-3HA-HAS1 HIS3 rpf2::RPF2-TAP URA3* [pRS315-*HAS1*]	This study
JWY9329	*MATa ura3-52 trp1-Δ101 lys2-801 his3-Δ200 leu2-Δ1 has1::GAL-3HA-HAS1 HIS3 rpf2::RPF2-TAP URA3* [pRS315-K92A]	This study
JWY9347	*MATa ura3-52 trp1-Δ101 lys2-801 his3-Δ200 leu2-Δ1 has1::GAL-3HA-HAS1 HIS3 rpf2::RPF2-TAP URA3* [pRS315-E197Q]	This study
JWY9330	*MATa ura3-52 trp1-Δ101 lys2-801 his3-Δ200 leu2-Δ1 has1::GAL-3HA-HAS1 HIS3 rpf2::RPF2-TAP URA3* [pRS315-S228A]	This study
JWY9331	*MATa ura3-52 trp1-Δ101 lys2-801 his3-Δ200 leu2-Δ1 has1::GAL-3HA-HAS1 HIS3 rpf2::RPF2-TAP URA3* [pRS315-T230A]	This study
JWY9332	*MATa ura3-52 trp1-Δ101 lys2-801 his3-Δ200 leu2-Δ1 has1::GAL-3HA-HAS1 HIS3 rpf2::RPF2-TAP URA3* [pRS315-H375E]	This study
JWY9348	*MATa ura3-52 trp1-Δ101 lys2-801 his3-Δ200 leu2-Δ1 has1::GAL-3HA-HAS1 HIS3 rpf2::RPF2-TAP URA3* [pRS315-CTD]	This study
JWY9357	*MATa ura3-52 trp1-Δ101 lys2-801 his3-Δ200 leu2-Δ1 has1::GAL-3HA-HAS1 HIS3 rpf2::RPF2-TAP URA3 rpl35B::RPL35B-3HA TRP1* [pRS315]	This study

JWY9359	*MATa ura3-52 trp1-Δ101 lys2-801 his3-Δ200 leu2-Δ1 has1::GAL-3HA-HAS1 HIS3 rpf2::RPF2-TAP URA3 rpl35B::RPL35B-3HA TRP1* [pRS315-K92A]	This study
JWY9365	*MATa ura3-52 trp1-Δ101 lys2-801 his3-Δ200 leu2-Δ1 has1::GAL-3HA-HAS1 HIS3 rpf2::RPF2-TAP URA3 rpl26A::RPL26A-3HA TRP1* [pRS315]	This study
JWY9362	*MATa ura3-52 trp1-Δ101 lys2-801 his3-Δ200 leu2-Δ1 has1::GAL-3HA-HAS1 HIS3 rpf2::RPF2-TAP URA3 rpl26A::RPL26A-3HA TRP1* [pRS315-K92A]	This study
JWY9391	*MATa ura3-52 trp1-Δ101 lys2-801 his3-Δ200 leu2-Δ1 has1::GAL-3HA-HAS1 HIS3 rpf2::RPF2-TAP URA3* [pRS315-Q69A]	This study
JWY9392	*MATa ura3-52 trp1-Δ101 lys2-801 his3-Δ200 leu2-Δ1 has1::GAL-3HA-HAS1 HIS3 rpf2::RPF2-TAP URA3* [pRS315-Q69A/K92A]	This study

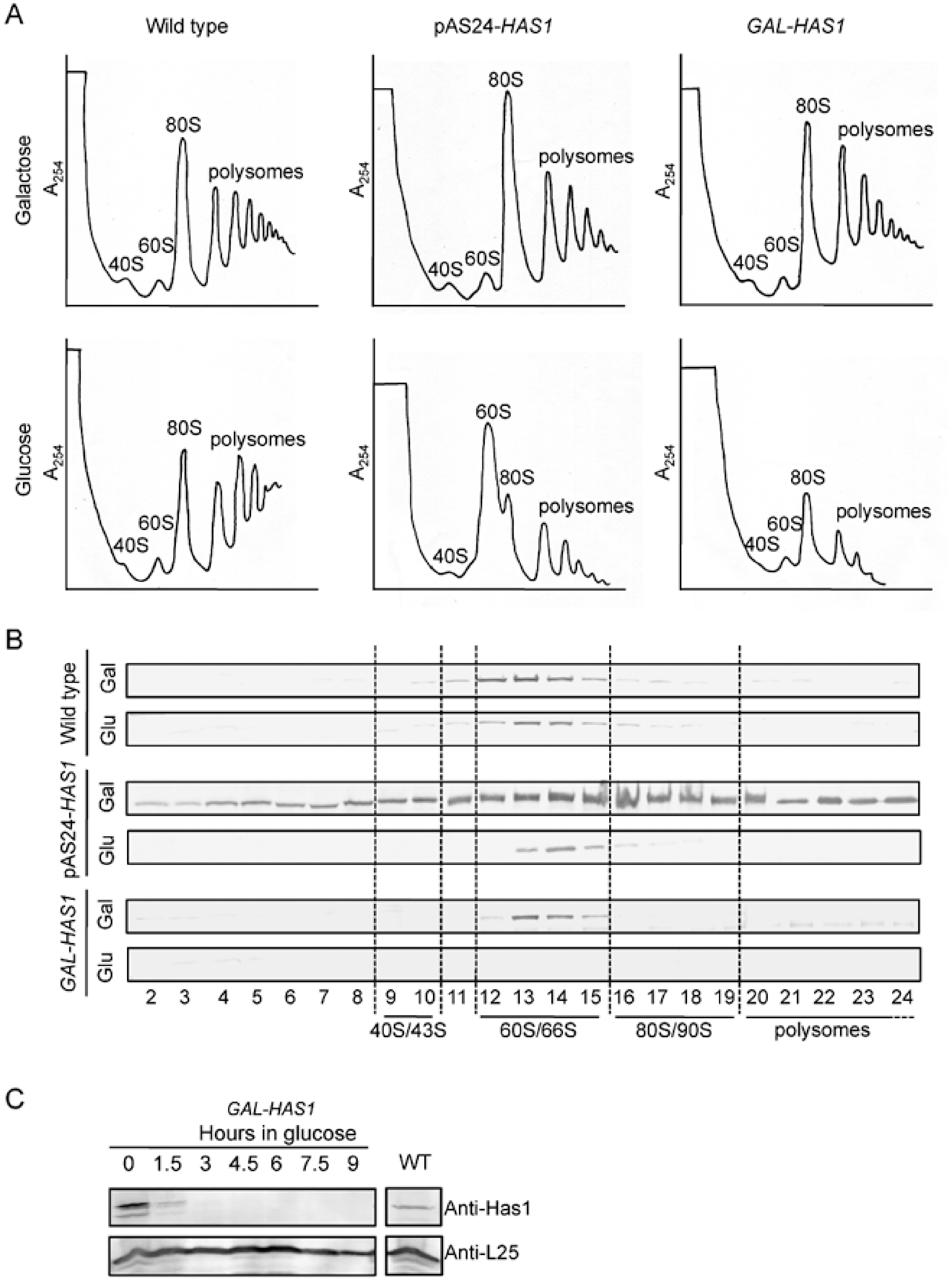
A
Wild type
pAS24-HAS1
GAL-HAS1
Galactose
Glucose
A254
40S
60S
80S
polysomes
B
Wild type
pAS24-HAS1
GAL-HAS1
Gal
Glu
2 3 4 5 6 7 8 9 10 11 12 13 14 15 16 17 18 19 20 21 22 23 24
40S/43S
60S/66S
80S/90S
polysomes
C
GAL-HAS1
Hours in glucose
0 1.5 3 4.5 6 7.5 9
WT
Anti-Has1
Anti-L25

Figure S1. Depletion of Has1 from 66S pre-ribosomes results in a 60S subunit deficit. (A) Cell lysates from wild type yeast and pAS24-*HAS1* and *GAL-HAS1* depletion strains grown in galactose- or glucose-containing medium were loaded onto sucrose gradients and subjected to centrifugation to resolve 40S, 60S and 80S monosomes and polysomes. (B) Sucrose gradient fractions were analyzed for sedimentation of Has1 by western blotting with the anti-Has1 antibody. The 40S peak contains 40S subunits and 43S pre-ribosomes, the 60S peak contains 60S subunits and 66S pre-ribosomes, and the 80S peak contains 80S monosomes and 90S pre-ribosomes. Fraction number is indicated below. (C) Cell lysates were analyzed for Has1 expression after time-course depletion by western blotting using anti-Has1. L25, loading control.

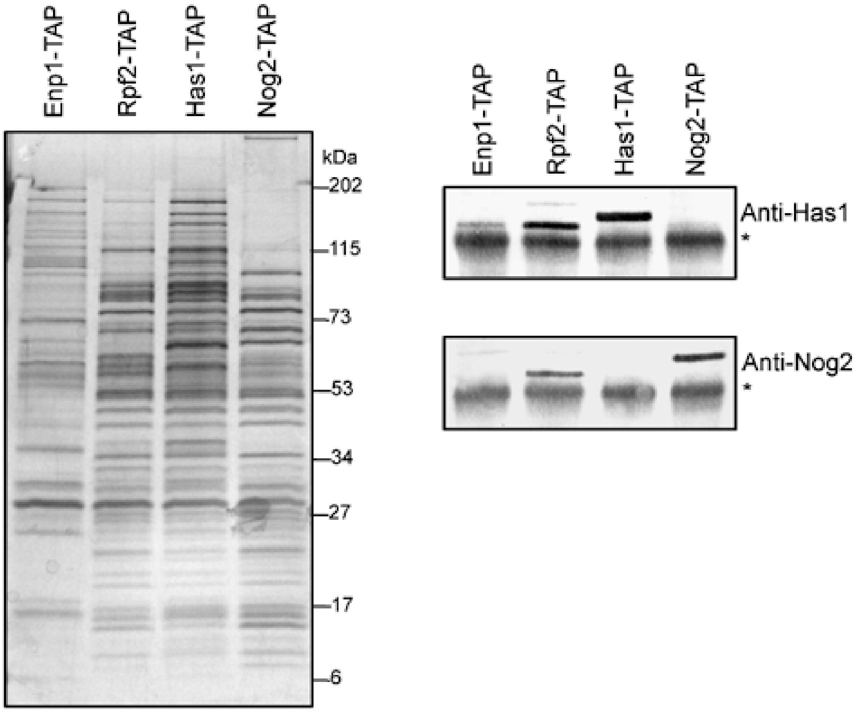

Figure S2. Has1 and Nog2 are mutually exclusive for association with 66S pre-ribosomes. Silver staining (left) and western blotting (right) of proteins that copurify with Has1- and Nog2-TAP reveal that the two proteins are not found in the same pre-ribosomal particles. Enp1-TAP is a control for 90S/43S pre-ribosomes and Rpf2-TAP is a control for 66S pre-ribosomes. Asterisks indicate IgG that was stripped from beads during purification.

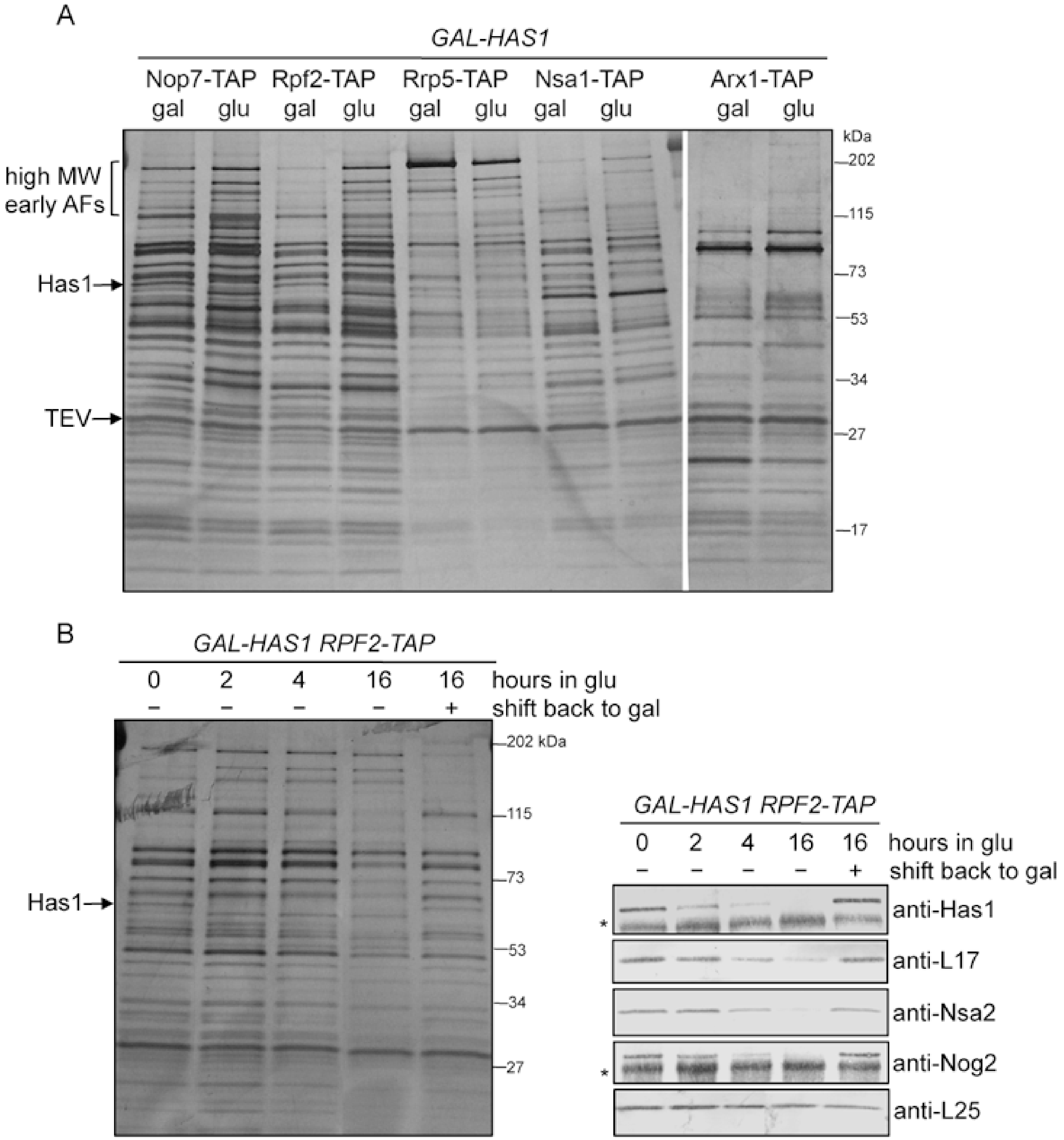

Figure S3. Pre-ribosomal particles are largely intact but shift to earlier intermediates after Has1 depletion. (A) Consecutive pre-ribosomal particles were purified using TAP-tagged assembly factors (Nop7, Rpf2, Rrp5, Nsa1, Arx1) in the presence (gal) and absence (glu) of the Has1 protein. Proteins found in successive pre-ribosomal intermediates were resolved by SDS-PAGE and visualized by silver staining. The locations of Has1 and TEV protease that were used to elute pre-ribosomes from IgG-coated beads are indicated. (B) Pre-ribosomes were purified from the *GAL-HAS1 RPF2-TAP* strain after shifting to glucose-containing media for the indicated period of time (hours in glu) followed by a shift back to galactose-containing media for four hours if indicated (−/+). A silver stained SDS-PAGE gel is shown (left), as well as western blots for individual proteins (right).

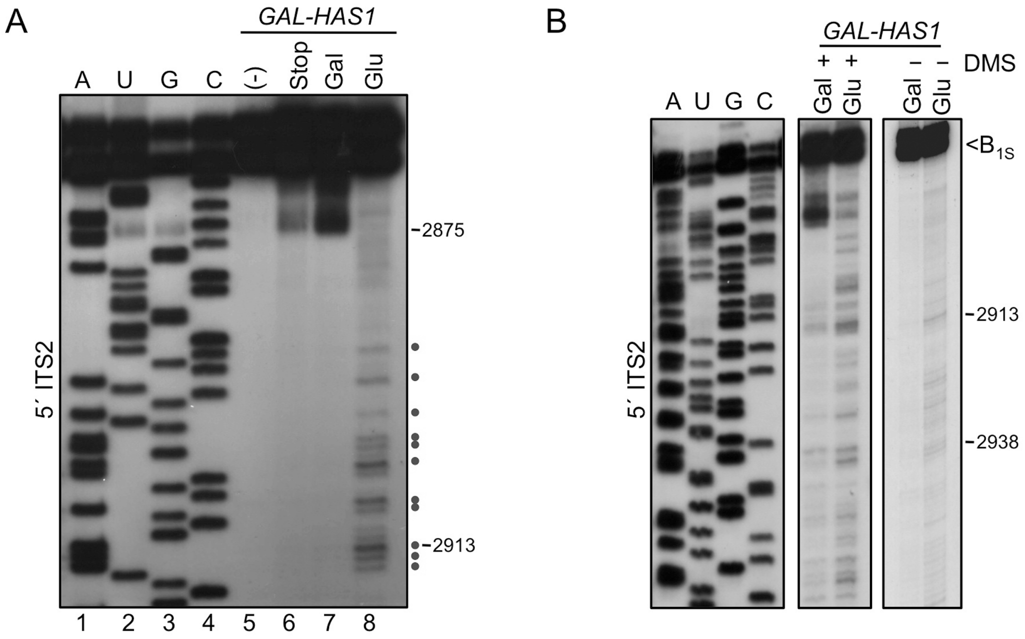

Figure S4. Nucleotides in 5.8S rRNA are more modified by DMS in the absence of Has1. (A) Better separation of nucleotides in 5.8S rRNA reveals additional residues that are more accessible to chemical modification in the absence of Has1. Primer extension of modified RNA was carried out as in Figure 6A using the 5´ ITS2 oligo and the sequencing gel was run longer to better resolve nucleotides 2875-2913. Nucleotides that become more modified in the absence of Has1 are indicated by grey circles. Nucleotide positions are indicated for 35S pre-rRNA. (B) The No DMS control is shown for the *GAL-HAS1* strain grown in galactose (Gal) and glucose (Glu) containing media. Primer extension was carried out with the 5´ ITS2 oligo and a region of 5.8S rRNA is shown.

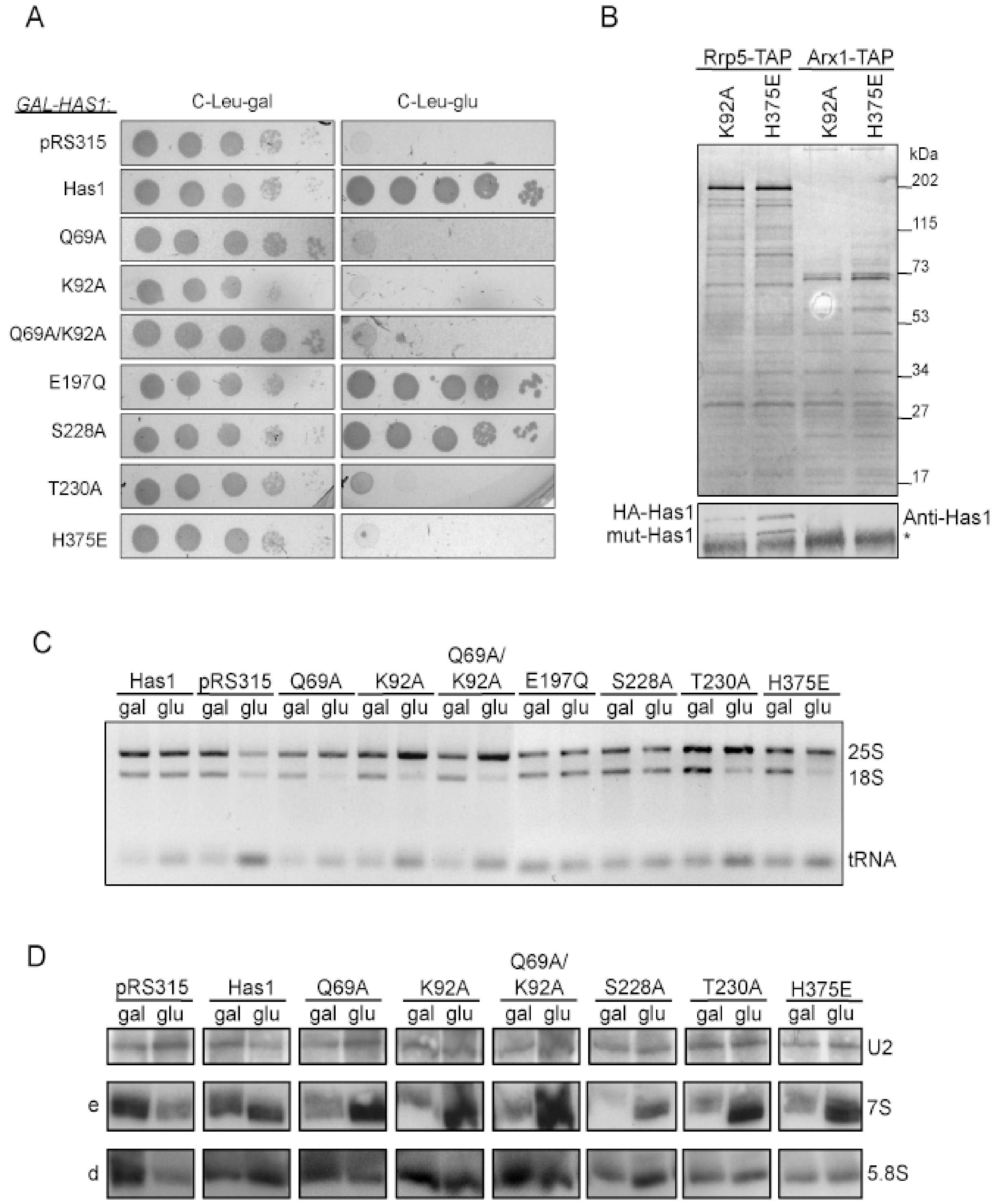

Figure S5. ATP-dependent activity of Has1 is essential for 18S rRNA production. (A) *LEU2*-marked plasmids containing no insert (pRS315), wild type or mutant *has1* alleles (left) were transformed into the *GAL-HAS1* yeast strain. Ten-fold serial dilutions of these strains were spotted onto galactose- (C-Leu-gal) or glucose (C-Leu) containing medium. (B) Plasmids bearing the K92A or H375E mutant versions of *Has1* were transformed into *GAL-HAS1* strains expressing Rrp5- or Arx1-TAP and assayed for association with pre-ribosomes by western blotting with the anti-Has1 antibody (bottom). The top band corresponds to the HA-tagged wild type copy of Has1 (HA-Has1) and the

bottom band corresponds to the untagged mutant version of Has1 (mut-Has1). The asterisk indicates IgG that was stripped from beads during purification. A silver stained gel of purified Rrp5- and Arx1-TAP particles is shown for comparison of loading (top). (C) Effects of *has1* mutations on 18S and 25S rRNA levels relative to tRNA levels were compared by agarose gel electrophoresis of cellular RNA. (D) Effects of *has1* mutations on cellular RNA were analyzed by northern blotting. Oligonucleotide probes (left) and RNAs detected (right) are indicated as in Figure 1A. U2 snRNA is the loading control. The same RNA samples were used as in Figure 7D.

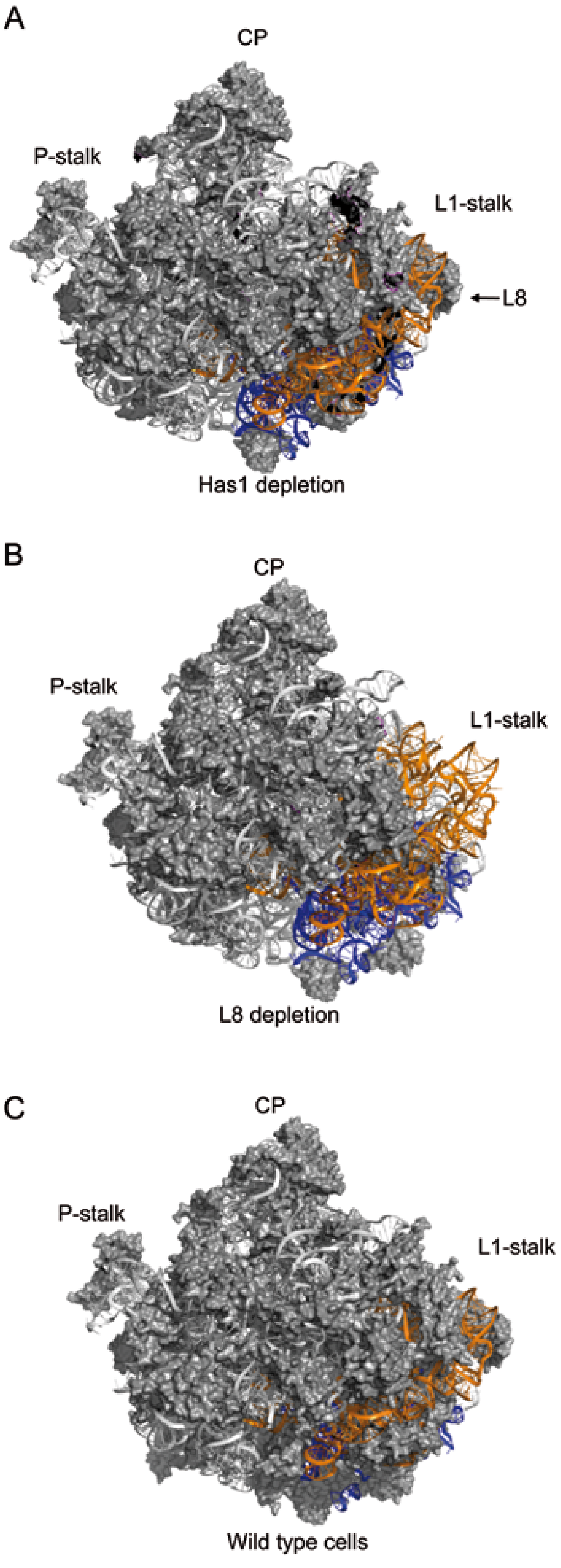
A
CP
P-stalk
L1-stalk
L8
Has1 depletion
B
CP
P-stalk
L1-stalk
L8 depletion
C
CP
P-stalk
L1-stalk
Wild type cells

Figure S6. The relative amounts of r-proteins bound to domain I of 5.8S/25S rRNA correlate with the extent of turnover of 27S pre-rRNA intermediates. (A) Pymol representation of the 60S ribosomal subunit is shown with r-proteins as dark grey spheres and rRNA as light grey ribbons. R-proteins that bind to domain I of the 5.8S/25S rRNA (orange ribbons) and domain III of the 25S rRNA (blue ribbons) are missing after Has1 depletion. Locations of the central protuberance (CP), P-stalk and L1-stalk are shown on the solvent accessible interface of the 60S subunit. (B) R-proteins L8, L13, L15 and L36 are also missing from 66S pre-ribosomes after depletion of r-protein L8, resulting in more rapid turnover of pre-rRNA intermediates than after Has1 depletion (31). (C) Wild type ribosomes are shown for comparison.

SUPPLEMENTARY EXPERIMENTAL PROCEDURES

Sucrose Gradients

Pre-ribosomes, ribosomes and polysomes were fractionated on 7%-47% (w/v) sucrose gradients as described previously (56) with a few modifications. Cycloheximide (5 mg) was added to 100 mL cultures 15 min before harvesting cells and 40 OD_{254} units of whole cell lysate were loaded onto gradients. A Teledyne ISCO Foxy R1 density gradient fractionator was used to monitor OD_{254} and separate fractions across the gradients.
